# Multimodal cortical and subcortical exercise compared with treadmill training for spinal cord injury

**DOI:** 10.1371/journal.pone.0202130

**Published:** 2018-08-09

**Authors:** Stephanie A. Martinez, Nhuquynh D. Nguyen, Eric Bailey, Denis Doyle-Green, Henry A. Hauser, John P. Handrakis, Steven Knezevic, Casey Marett, Jennifer Weinman, Angelica F. Romero, Tiffany M. Santiago, Ajax H. Yang, Lok Yung, Pierre K. Asselin, Joseph P. Weir, Stephen D. Kornfeld, William A. Bauman, Ann M. Spungen, Noam Y. Harel

**Affiliations:** 1 James J. Peters VA Medical Center, Bronx, New York, United States of America; 2 New York Institute of Technology, Old Westbury, New York, United States of America; 3 Icahn School of Medicine at Mount Sinai, New York, New York, United States of America; 4 University of Kansas, Lawrence, Kansas, United States of America; University of Illinois at Urbana-Champaign, UNITED STATES

## Abstract

**Background and purpose:**

Spared fibers after spinal cord injury (SCI) tend to consist predominantly of subcortical circuits that are not under volitional (cortical) control. We aim to improve function after SCI by using targeted physical exercises designed to simultaneously stimulate cortical and spared subcortical neural circuits.

**Methods:**

Participants with chronic motor-incomplete SCI enrolled in a single-center, prospective interventional crossover study. Participants underwent 48 sessions each of weight-supported robotic-assisted treadmill training and a novel combination of balance and fine hand exercises, in randomized order, with a 6-week washout period. Change post-intervention was measured for lower extremity motor score, soleus H-reflex facilitation; seated balance function; ambulation; spasticity; and pain.

**Results:**

Only 9 of 21 enrolled participants completed both interventions. Thirteen participants completed at least one intervention. Although there were no statistically significant differences, multimodal training tended to increase short-interval H-reflex facilitation, whereas treadmill training tended to improve dynamic seated balance.

**Discussion:**

The low number of participants who completed both phases of the crossover intervention limited the power of this study to detect significant effects. Other potential explanations for the lack of significant differences with multimodal training could include insufficient engagement of lower extremity motor cortex using skilled upper extremity exercises; and lack of skill transfer from upright postural stability during multimodal training to seated dynamic balance during testing. To our knowledge, this is the first published study to report seated posturography outcomes after rehabilitation interventions in individuals with SCI.

**Conclusion:**

In participants with chronic incomplete SCI, a novel mix of multimodal exercises incorporating balance exercises with skilled upper extremity exercises showed no benefit compared to an active control program of body weight-supported treadmill training. To improve participant retention in long-term rehabilitation studies, subsequent trials would benefit from a parallel group rather than crossover study design.

## Introduction

Most spinal cord injuries (SCI) spare a portion of axonal fibers at the injury level [1,2]. Fibers of subcortical pathways such as the reticulospinal and propriospinal tracts make up a significant portion of spared circuitry that can mediate substantial functional recovery [3,4]. Subcortical circuits connect to many of the same spinal motor neurons to which corticospinal circuits connect [5–8]. Corticospinal fibers also make collateral connections to reticulospinal and other descending subcortical circuits [7,9]. Experiments in animals have demonstrated the potential for alternate or detour connections between cortical, subcortical, and spinal circuits to mediate recovery after SCI [10–13].

Our group has previously applied targeted physical exercises that are designed to facilitate detour connectivity by repetitively and simultaneously stimulating cortical and spared subcortical circuits. This multimodal (MM) exercise strategy combines postural tasks (which activate subcortical circuits) with fine motor tasks (which activate cortical circuits). In animal models and non-disabled human volunteers, we found that compared with exercises stimulating cortical or spinal circuits alone, MM exercises promoted improved recovery from central nervous system injury and increased corticospinal neurotransmission [14–16].

Robotic-assisted weight-supported treadmill exercise (TM) is an established form of physical rehabilitation that is associated with positive clinical outcomes in participants with incomplete SCI [17–20]. Treadmill training largely targets spinal locomotor central pattern generator circuits [21,22]. However, it requires expensive equipment and space that is generally available only in institutional settings. Based on our earlier findings, we initiated a clinical trial in humans with chronic SCI designed to compare the effects of our novel multimodal exercise program to that of treadmill training (TM). We hypothesized that compared with TM, MM exercises would significantly increase volitional lower extremity motor scores and corticospinal neurotransmission.

## Methods

### Participants

Participants between the ages of 21 and 65 with motor incomplete SCI (as determined by the International Standards for Neurological Classification of SCI (ISNCSCI)), or volitional strength of at least 1/5 according to ISNCSCI in two or more key lower extremity muscles, with ≥1 year duration of injury were recruited. Enrollment occurred between February 2013 and May 2016. Initially, only participants with thoracic SCI were included; entry criteria were later expanded to include participants with injury level between C2-T12 who had at least anti-gravity strength in the deltoids, biceps, and triceps muscles. Exclusion criteria included significant neurological or coronary artery disease, severe osteoporosis, severe joint stiffness, or excessive risk of transcranial magnetic stimulation (epilepsy, prior intracranial hemorrhage, amphetamine usage, and other factors that increase seizure risk). A full list of inclusion and exclusion criteria is provided on ClinicalTrials.gov, where this study was registered (https://clinicaltrials.gov/ct2/show/NCT01740128). Subjects provided written informed consent. All procedures and data analysis occurred at the James J. Peters Veterans Affairs Medical Center, with approval by the Institutional Review Board of the Bronx VA Medical Center Research & Development Program (151) (Protocol #01407). All applicable institutional and governmental regulations concerning the ethical use of human volunteers according to the principles of the Declaration of Helsinki were followed during the course of this research.

### Design

**Fig 1:**
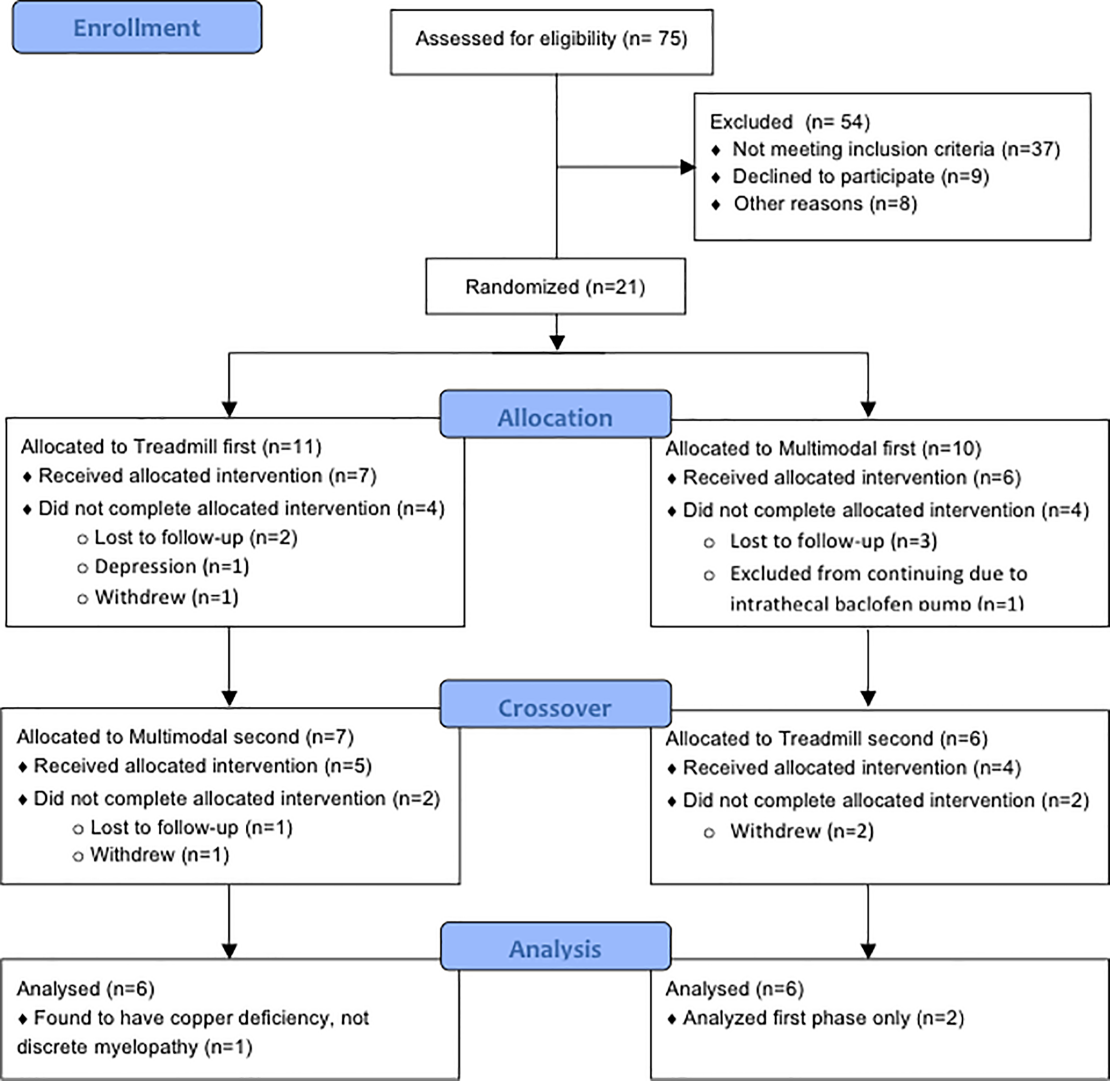
CONSORT flow diagram.

This study was a single-group, partially blinded crossover trial (Fig 1). Each phase consisted of 48 sessions of one intervention followed by a washout period of at least 6 weeks. TM or MM intervention order was randomly assigned using Research Randomizer (www.randomizer.org). An otherwise unaffiliated staff member revealed the intervention allocation after study personnel obtained informed consent from each newly enrolled participant. Three to five sessions per week were scheduled. For subject convenience, up to two sessions were performed during a single visit, with a rest period of at least 30 minutes between sessions. Outcome assessments were performed at baseline and within one week of completing an intervention. An expert evaluator blinded to intervention assessed the primary clinical outcome (lower extremity motor score). A follow-up evaluation was planned for 6 weeks after intervention completion. With expected dropout rate of 25% and effect size of 1 based on prior manuscripts reporting electrophysiological outcomes [23,24], enrollment of 24 participants was calculated to provide power ≥0.83 to reject the null hypothesis on a two-tailed independent-sample t-test with alpha of 0.05.

### Exercises

#### General

Sessions lasted 30 minutes not including setup, with 1–2 minute rest periods at least every 10 minutes. Vital signs (brachial cuff blood pressure, heart rate recorded by Dinamap V100) and rating of perceived exertion (RPE) via the Borg Scale [25] were recorded at least twice per session. Exercise task difficulty was adjusted as detailed below to achieve a desired range of RPE between 11 to 15 (out of 20). Study personnel continuously monitored and frequently questioned participants for any adverse symptoms. Body weight support (BWS) for both interventions was provided by the overhead harness of the Lokomat system (Hocoma). BWS was set to 60% of body weight initially, and then gradually reduced as tolerated. Note that even subjects with complete paraplegia can passively support 40% or more of their own body weight in the upright position [26–28]. Several participants who reached independent weight support still wore the harness for safety.

#### Treadmill exercise (TM)

Participants walked on a robotic-assisted treadmill (Lokomat, Hocoma) at initial speeds of 1–1.5 km/h. Speed was gradually increased as tolerated to a maximum of 3.2 km/h. The Lokomat’s built-in guidance force (amount of assistance to reach a predefined gait kinematic pattern) was also gradually reduced as tolerated. Participants were reminded to swing their arms while walking.

#### Multimodal exercise (MM)

**Fig 2:**
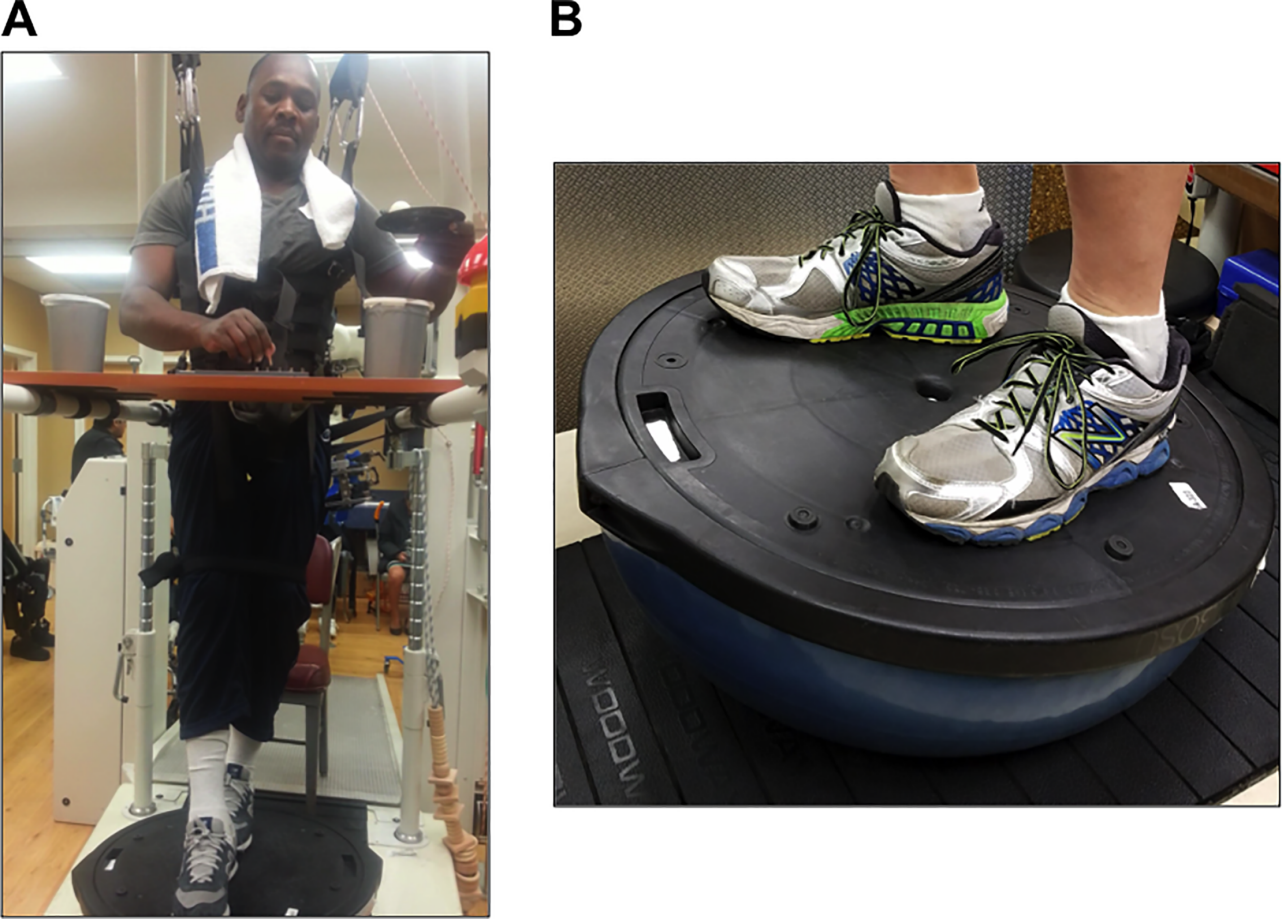
Multimodal (MM) exercise paradigm. MM involved balance exercises plus simultaneous fractionated hand exercises targeted at the corticospinal tract. Participants’ feet were placed on either the flat or convex side of a semi-spherical balance platform (Bosu™). To increase challenge, participants were intermittently asked to perform tandem stance (A), or study personnel applied external perturbations. During balance exercise, participants performed a variety arm or hand manipulations that involve precision or power movements, such as placing pegs into a grooved pegboard, maintaining a ping-pong ball on a small handheld plastic dish (A); and other tasks. Tasks were varied every one to three minutes to maintain participant interest. Overhead partial body weight support was provided at all times.

Participants performed simultaneous balance and skilled upper extremity exercises. In addition to partial body weight support using the Lokomat harness, study personnel provided manual stabilization and perturbation as necessary. *Balance (subcortical) component*: Participants’ feet were placed on a semi-spherical balance platform (Bosu™, 63.5 cm diameter, 23 cm height). Either the flat or convex sides of the balance ball were utilized as the standing surface, but generally, the flat side was in contact with the feet, and the convex side was in contact with the ground (Fig 2). Participants were instructed to keep the balance surface as stable as possible. To increase level of difficulty in either orientation, study personnel manually applied external perturbations to either the balance ball or the participants’ trunks. *Fine upper extremity (corticospinal) component*: During balance exercise, participants performed a variety of skilled arm or hand manipulations, either unimanually or bimanually. All tasks were designed to require movements that engage corticospinal circuits [29,30]. Tasks were varied every few minutes to maintain participant interest. Tasks included inserting different-sized coins into slots oriented at different angles; tightening or loosening screws from a board; picking up playing cards or paper clips off a flat surface; performing a skilled pegboard task (Lafayette Instruments); guiding a loop over an alarmed irregularly curved coil; typing numbers on a keypad; threading beads on a string; inserting long-handled keys into custom slots that required forearm supination; maintaining a ping-pong ball on a small handheld plastic dish; and others (Fig 2; S5 File).

### Lower extremity motor score (LEMS)

Manual assessment of muscle strength in both legs was performed at 5 key myotomes (L2 = hip flexion; L3 = knee extension; L4 = ankle dorsiflexion; L5 = toe dorsiflexion; S1 = ankle plantarflexion) according to the ISNCSCI [31]. Muscles were scored on a scale of 0 to 5, resulting in possible LEMS ranging from 0 to 50.

### Berg Balance Scale (BBS)

Most participants were unable to attempt the standing portions of the Berg Scale [32]. Therefore, the ‘Sitting with back unsupported’ subsection was compared. Participants sat on a cushioned adjustable chair without back support, with hip, knee, and ankle angles at approximately 90 degrees, and arms folded across the chest for up to two minutes.

### Computerized posturography

A wooden block (46 cm W x 43 cm L x 31 cm H) was centered on a Smart Equitest (Natus) force plate, with an overlying foam pad (13 cm thick) for subject comfort and skin protection [33]. Participants were seated without back support, with hip, knee, and ankle angles at approximately 90 degrees, and arms folded across the chest. In the Limits of Stability test, participants were instructed to shift their center of gravity (represented as an avatar in real time) toward eight surrounding targets on a computer monitor. Key measures were endpoint and maximal excursion of the center of gravity (EPE and MXE), and directional control (DCL) of intended movements. In the Clinical Test of Sensory Integration on Balance, participants were asked to maintain static upright posture for 10 seconds each under four conditions: 1) arms crossed over the chest with eyes open; 2) arms over the chest with eyes closed; 3) arms outstretched forward with eyes open; 4) arms outstretched forward with eyes closed. Each condition was tested three times. COP angular displacement in the mediolateral and anteroposterior planes was averaged over 10 seconds per trial. The primary static outcome measure of sway velocity represents the difference in average sway (degrees per second) in the eyes-closed versus the eyes-open arms crossed condition.

### Modified Ashworth Scale (mAS)

The mAS was assessed at the knee extensors on both sides using six scoring levels (0,1,1+,2,3,4), where 0 is defined as no increase in muscle tone and 4 is defined as the affected part rigid in flexion or extension [34]. For analysis, mAS scores were transformed into a 0–5 scale and averaged between the left and right legs.

### Spinal Cord Injury Spasticity Evaluation Tool (SCI-SET)

This survey was used to evaluate how spasticity impacted a person’s activities of daily living over the course of the previous 7 days [35].

### McGill Pain Questionnaire (short form)

This survey was used to track whether any neural plastic changes resulted in adverse effects on neuropathic pain [36].

### Gait speed and seated step test

The 10-meter walk test was administered using standard measures (walk at maximal safe speed, with or without assistive devices; 2-meter lead-in; average of three repetitions). For the 10-second seated step test [37], participants were seated with their hips and knees flexed to 90 degrees, then asked to lift one foot entirely off the ground and place it back down again. The average number of steps taken during three 10-second trials with each foot was recorded.

### Surface EMG

Adhesive snap dual surface electrodes (Natus) were applied to the bellies of the tibialis anterior and soleus muscles. Recordings were collected using a Viking Select system (Natus) or a Motion Lab Systems system with wired electrodes. Samples were acquired at a rate of 5,000 Hz. Adverse events during electrophysiological testing were collected with a questionnaire developed by an international expert consensus panel on TMS safety [38].

### Peripheral responses

Responses to external stimulation were recorded in the resting supine position, with the knees resting on a foam roll and the ankles in neutral position. Electrical stimuli were delivered using a Grass S88 dual-output stimulator (Natus) or a DS7A stimulator (Digitimer). The peroneal nerve was stimulated at the fibular head with 0.2 ms pulses at supramaximal intensity to define the tibialis anterior (TA) maximal compound motor action potential (Mmax) as well as to elicit F-waves in the antidromic orientation. The tibial nerve was stimulated in the popliteal fossa with 1.0 ms pulses at a range of intensities to determine the threshold and slope of the H-reflex recruitment curve, and the maximal soleus M-wave amplitude (Mmax). A minimum of 5 seconds elapsed between each tibial nerve pulse.

### Transcranial magnetic stimulation

A MagPro system (Magventure) with 80mm winged coil (D-B80) was centered over the leg motor cortex hotspot for maximal tibialis anterior response (usually 2 cm lateral to the vertex). The coil was maintained in position with a multijointed mechanical arm (MagVenture). Subjects wore a white cloth TMS cap. The ‘hotspot’ was marked in permanent ink on the cap, which was carefully repositioned and reused for each subject across testing sessions. Assessors vigilantly checked coil, cap, and head positioning during testing. Resting motor threshold (RMT) was determined as the percent of maximal stimulator output required to elicit a potential of at least 25μV in 5 out of 10 repetitions. The 25μV rather than 50μV threshold was used due to the inherently lower capability to evoke responses in the leg muscles in comparison to hand muscles. Motor evoked potential (MEP) amplitudes were averaged across 5 repetitions per intensity. To account for changes in electrode placement and conductance over different testing sessions, MEPs were normalized to that session’s peripherally evoked Mmax [39].

### Soleus H-reflex facilitation by transcranial magnetic stimulation

H-reflex stimuli were delivered via surface electrodes in the popliteal fossa with intensity set to elicit an H-reflex of 10–20% of Mmax [40]. TMS pulses were delivered at 80–90% of tibialis anterior resting motor threshold (or 80–90% of maximum stimulator output if TA RMT was unobtainable), at interstimulus intervals (ISI) of 0–120 ms prior to the H-reflex stimulus. Sets of ISI combinations were delivered in pseudorandom order. A minimum of 10 seconds elapsed between each pulse. Participants were instructed to mentally focus on plantarflexing the targeted ankle during H-reflex facilitation assessment. TMS-conditioned soleus H-reflex amplitude (average over 5 repetitions) was compared to unconditioned H-reflex amplitude to determine the percent facilitation at each ISI. Results at ISI between 0 and 20 ms were grouped into ‘short-interval facilitation’, whereas results at ISI between 60–120 ms were grouped into ‘long-interval facilitation’ [41]. H-reflex amplitudes were averaged between both legs within each subject, except for two participants who had elicitable H-reflexes on only one side.

### Statistics

The a priori primary clinical outcome was post-intervention change in lower extremity motor score. The intended a priori primary neurophysiological outcome was post-intervention change in the amplitude of the tibialis anterior motor evoked potential (TA MEP). However, only two participants demonstrated consistent TA MEPs at baseline. Therefore, soleus H-reflex facilitation was employed as the primary neurophysiological outcome measure. Due to the small sample size, median and interquartile range are reported for all outcomes. The data were analyzed using linear mixed modeling with intervention (TM vs MM; a repeated measure factor) and order (TM-first or MM-first; a between-subjects factor) modeled as fixed effects. The dependent variable was the change score (post—pre) for the respective measurements. The underlying covariance structure was compound symmetry.

Missing values for specific outcome tests (highlighted in S4 File) were not imputed. Carryover effect among participants who completed both intervention phases was tested by subtracting changes during the second intervention from changes during the first intervention for each subject, then performing an unpaired t-test between subjects who performed TM first and subjects who performed MM first. Significance (including Bonferroni correction) was set at p<0.025 for primary outcomes and p<0.005 for secondary outcomes. Post hoc power calculations for the outcomes of short-interval H-reflex facilitation and lower extremity motor score were calculated from the fixed effects of Intervention using G*Power version 3.1.9.3. Microsoft Excel, IBM SPSS, and the lme4 package in R statistical software were used for all other analyses.

## Results

21 of a planned 24 participants were enrolled between February 2013 and May 2016. Enrollment completed when funding expired. Only 13 participants completed at least one intervention phase of the study, and 9 completed both phases of the study (Fig 1). We report data from participants who completed at least one intervention (demographics detailed in Table 1). One subject was found after study entry to have copper-deficient myeloneuropathy rather than a discrete spinal injury, so his data was not included. One subject who had been classified as motor incomplete SCI in another research study within our center was reclassified as motor-complete SCI after enrolling in our study. However, due to the presence of three other subjects with baseline LEMS of three or less in the small subject sample, he was retained in our study. There were no serious adverse events during the study. Several participants reported mild adverse events such as lightheadedness or skin abrasions.

**Table 1:** Subject demographic characteristics.

**MF**	**Age**	**Trauma/NT**	**DOI**	**Level**	**AIS**	**Interventions**
**F**	**36**	**NT**	**18**	**T2**	**C**	**MM**
**M**	**45**	**T**	**5.5**	**T8**	**A**	**TM, MM**
**M**	**50**	**NT**	**1**	**T9**	**C**	**TM, MM**
**M**	**23**	**T**	**4**	**T10**	**D**	**TM, MM**
**M**	**46**	**T**	**23**	**T4**	**C**	**MM, TM**
**M**	**41**	**T**	**1**	**T8**	**C**	**MM**
**M**	**42**	**T**	**4**	**T1**	**B**	**TM, MM**
**F**	**37**	**T**	**11**	**T11**	**C**	**TM**
**M**	**40**	**T**	**4**	**C6**	**D**	**TM**
**M**	**51**	**T**	**16**	**C8**	**D**	**MM, TM**
**M**	**29**	**T**	**2.5**	**C8**	**C**	**MM, TM**
**F**	**44**	**T**	**5**	**T10**	**C**	**MM, TM**

M = male. F = female. T = traumatic. NT = non-traumatic. DOI = duration of injury (years). AIS = ASIA Impairment Scale. TM = treadmill intervention. MM = multimodal intervention. Subjects completed 48 sessions of the listed intervention(s). Interventions are listed in order of completion.

All outcomes are summarized in Table 2. No statistically significant differences between or within interventions were found for any outcome. Raw data and statistical tabulations are detailed in S4 File.

**Table 2:** Intervention effects on study endpoints.

			LEMS	Short Facil	Long Facil	Berg S3	Sway	EPE	MXE	DCL	mAsh	10 MWT	Seated Steps	SCI-SET	SD	AD
**Post**	**TM**	n	10	8	8	8	9	9	9	9	9	3	4	6	7	7
		**Median**	**0.0**	**-5.9**	**-2.1**	**0.0**	**0.0**	**8.9**	**5.1**	**9.0**	**0.0**	**0.1**	**-1.3**	**-0.5**	**0.0**	**0.0**
	** **	IQR	-2.0, 0.8	-13.8,2.4	-11.2,5.0	0.0, 0.0	-0.1, 0.2	-5.1, 17.1	1.8, 23.8	-1.4, 18.5	-0.5, 0.0	0.1,0.2	-2.4, -1.1	-1.0, 0.0	-2.0, 0.0	-0.0, 0.0
	**MM**	n	9	7	7	10	10	10	10	10	5	1	4	7	9	9
		**Median**	**0.0**	**2.4**	**-6.7**	**0.0**	**-0.2**	**2.3**	**1.6**	**-0.4**	**0.0**	**0.0**	**3.3**	**0.0**	**0.0**	**0.0**
		IQR	-2.0, 2.0	-0.6, 12.1	-27.8, 2.0	0.0, 0.0	-0.3, 0.0	-1.2, 12.3	-3.2, 8.5	-2.4, 1.8	0.0, 0.1	0.0, 0.0	2.0, 4.6	-4.0, 9.0	0.0, 4.0	0.0, 1.0
**6 Wk**	**TM**	n	7			6	8	8	8	8	6	3	3	5	6	6
		**Median**	**0.0**			**0.0**	**0.1**	**0.7**	**-3.1**	**1.5**	**0.0**	**0.1**	**1.3**	**5.0**	**0.0**	**0.0**
		IQR	-1.0, 1.5			0.0, 0.0	-0.2, 0.3	-4.9, 9.3	-5.0, 8.3	0.2, 5.2	-0.4, 0.0	0.0, 0.2	0.8, 2.7	3.0, 7.0	-0.8, 1.5	-0.8, 0.0
	**MM**	n	6			6	6	6	6	6	4	1	2	4	5	5
		**Median**	**0.0**	** **	** **	**0.0**	**-0.3**	**11.3**	**3.6**	**4.9**	**-0.3**	**0.0**	**1.7**	**3.0**	**-2.0**	**0.0**
		IQR	-1.5, 0.0			0.0, 0.8	-0.5, -0.1	3.4, 13.8	1.4, 7.5	1.5, 8.3	-0.8, 0.0	0.0, 0.0	1.3, 2.1	-3.3, 9.0	-3.0, 0.0	0.0, 0.0

No statistically significant differences between or within interventions were found for any outcome. Post = immediately post-intervention. 6 Wk = 6-week follow-up. TM = treadmill intervention. MM = multimodal intervention. n = number of subjects completing assessment. IQR = interquartile range. LEMS = lower extremity motor score. Short Facil = soleus H-reflex facilitation by subthreshold TMS between 0–20 ms interstimulus interval. Long Facil = soleus H-reflex facilitation by subthreshold TMS at 60 ms or more interstimulus interval. Berg S3 = score on the “Sitting with back unsupported subsection of Berg Balance Scale”. Sway = postural sway during upright sitting in eyes-closed relative to eyes-open condition. EPE = endpoint excursion. MXE = maximal excursion. DCL = directional control. mAsh = modified Ashworth. 10MWT = 10-meter walk test (m/s). SCI-SET = Spinal Cord Injury Spasticity Evaluation Tool. SD = sensory domain of McGill Pain Questionnaire. AD = affective domain of McGill Pain Questionnaire.

### Lower extremity motor score

**Fig 3:**
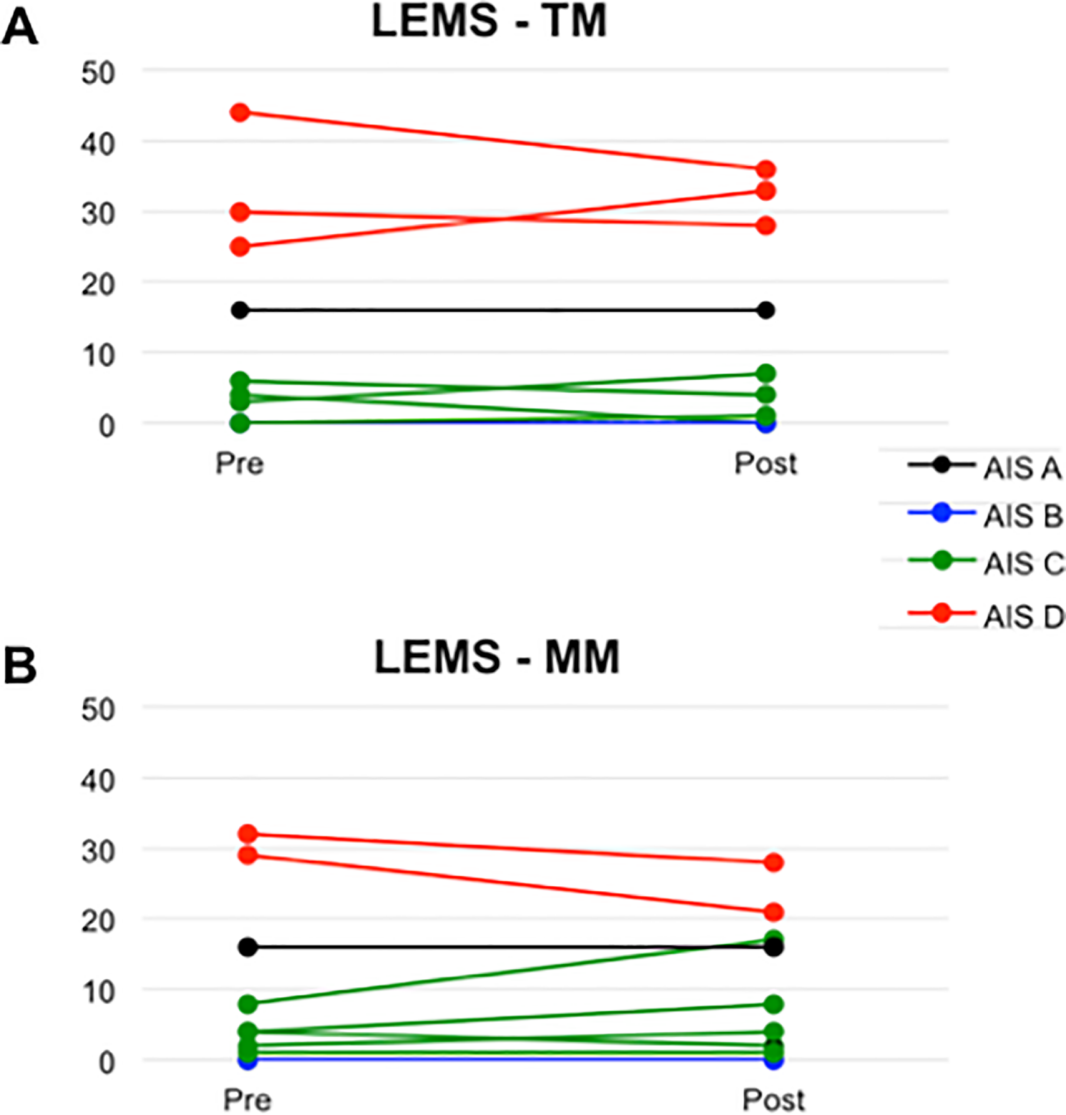
Change in Lower Extremity Motor Score (LEMS). Pre- and post-intervention data shown for each subject. **A**, Multimodal; **B**, Treadmill. Red lines indicate subjects with baseline ASIA Impairment Scale (AIS) Grade D. Green lines indicate subjects with baseline AIS Grade C. Blue line indicates subject with baseline AIS Grade B. Black line indicates subject with baseline AIS Grade A (and significant zone of partial preservation).

Participants had a wide range of LEMS at baseline (0–44). After TM, three participants demonstrated improvement in LEMS, three showed no change, and four deteriorated. After MM, three participants demonstrated improvement in LEMS, three showed no change, and three deteriorated (Fig 3). One post-MM participant completed all post-testing except for the INSCSCI examination. There was no significant difference in the rates or degree of LEMS improvement between the two interventions. The post-hoc observed power was 0.051.

### Soleus H-reflex facilitation

**Fig 4:**
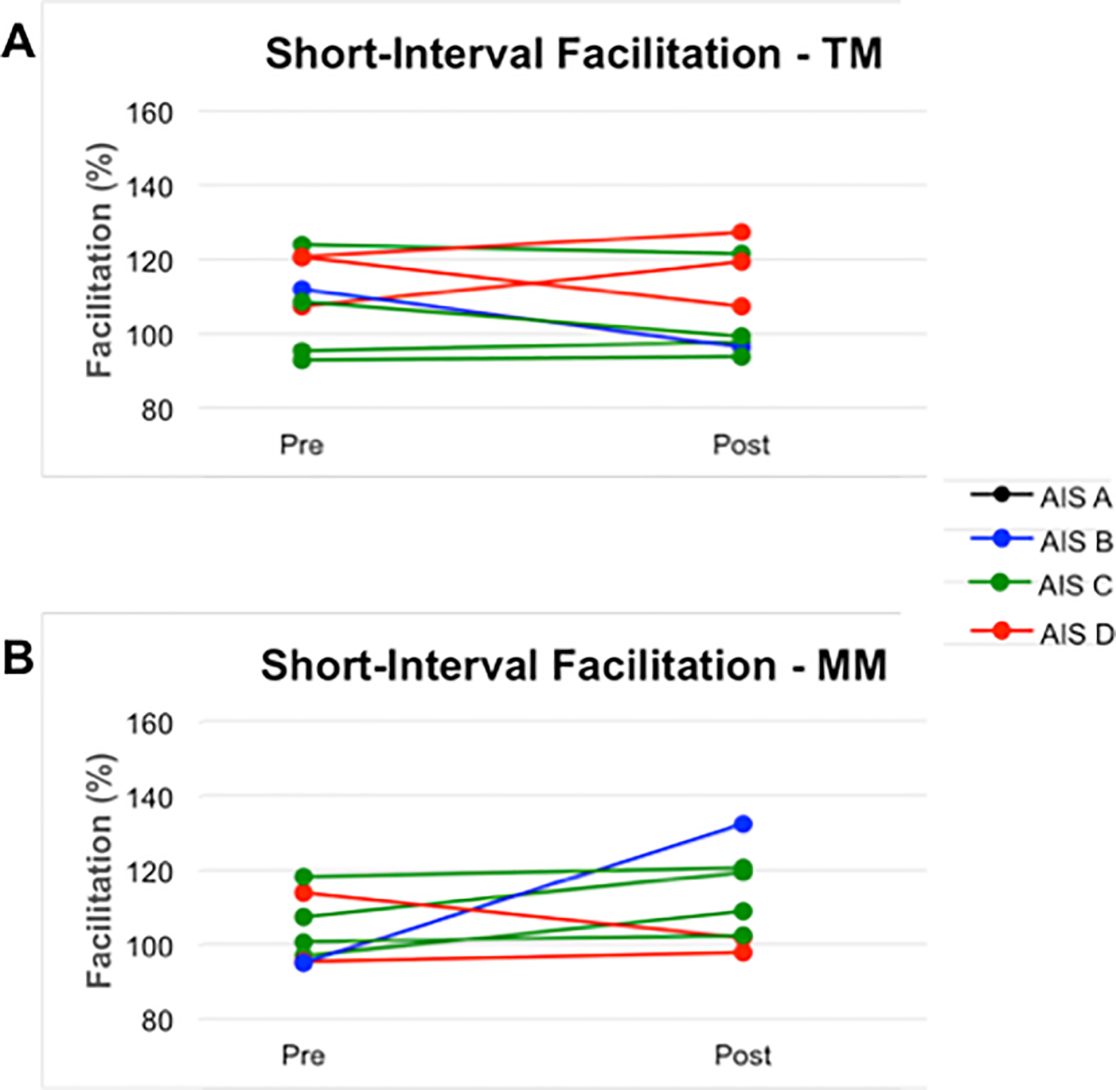
Change in short-interval (0–20 ms) soleus H-reflex facilitation by subthreshold transcranial magnetic stimulation. Pre- and post-intervention data shown for each subject. **A**, Multimodal; **B**, Treadmill. Red lines indicate subjects with baseline ASIA Impairment Scale (AIS) Grade D. Green lines indicate subjects with baseline AIS Grade C. Blue line indicates subject with baseline AIS Grade B. Black line indicates subject with baseline AIS Grade A (and significant zone of partial preservation).

After TM, three participants demonstrated an increase in short—interval facilitation (interstimulus interval 0–20 ms), and five showed a decrease. After MM, five participants demonstrated an increase in short-interval facilitation, and two showed a decrease (Fig 4). There was no statistically significant difference in the degree of H-reflex facilitation change between the two interventions, although MM tended to result in larger improvement than TM in short-interval facilitation (p = 0.053 before Bonferroni correction). The post-hoc observed power was 0.211.

### Secondary outcomes

#### Seated balance

Most participants were able to sit independently at baseline, achieving the highest possible score on the Sitting unsupported portion of the Berg Scale, thereby establishing a ceiling effect. Computerized posturography assessments were more sensitive for detecting change after intervention [33]. Contrary to our hypothesis, dynamic seated reaching tests, especially maximal excursion and directional control, showed a slight trend (p value 0.26 and 0.29, respectively, before Bonferroni correction) toward more improvement after TM than MM.

#### Spasticity

After TM, five participants demonstrated no change in knee extensor mAsh, and four showed a decrease. After MM, three participants demonstrated no change in mAsh, and 2 showed an increase. The SCI-SET survey was initiated partway through the study, so fewer subjects completed pre- and post-testing. SCI-SET score changes were minimal on the individual and group level.

#### McGill Pain Questionnaire (short form)

Median sensory and affective domain scores did not change after either intervention.

#### Gait speed and seated step test

Data was successfully collected from only a few participants with ambulatory capability and is shown in Table 2. After TM, one of four subjects increased the number of seated steps. After MM, four of four subjects increased the number of seated steps. However, these results did not reach statistical significance (step test p = 0.076 before Bonferroni correction).

#### Effects of order and carryover

Of the eight included subjects who completed both phases of the study, four each performed TM first or MM first. Order, included as a between-subjects factor in the multilevel analysis of variance, did not significantly affect any primary or secondary outcome. Carryover effect (defined as change during the second intervention subtracted from change during the first intervention within each subject) for primary outcomes was compared using unpaired t-tests between subjects who performed TM first and subjects who performed MM first. There were no statistically significant carryover effects.

## Discussion

Our novel targeted approach aims to improve the specificity and efficacy of exercise rehabilitation by simultaneously activating corticospinal and subcortical circuits. The corticospinal tract makes collateral connections with subcortical pathways as it passes through the brainstem. Subcortical circuits are often spared after SCI [3]. Based on our prior studies, we speculated that repetitive, synchronized activation of cortical and subcortical circuits through physical exercises could improve recovery by strengthening collateral corticobulbar synapses that could mediate functional detour connections between cortical and spared spinal circuits [6,13,42].

We tested a multimodal exercise paradigm combining fine hand tasks with postural stabilization exercises. Hand tasks such as the ones used in this study require fractionated finger movements and forearm supination, which activate corticospinal circuits [9,43]. Postural instability activates multiple subcortical areas, including reticulospinal, vestibulospinal, propriospinal, basal ganglia, and cerebellar pathways [44,45]. The MM regimen does not require a treadmill, robotic exoskeleton, or extensive manual assistance–therefore, MM would be simpler and less expensive than body weight-supported treadmill training to implement as a treatment modality.

In a small population with chronic incomplete SCI, we did not observe a significant difference in any outcome between MM and TM training in a 48-session crossover study. Although TM training (with manual or robotic assistance) has repeatedly been shown to improve stepping and locomotor function in individuals with chronic SCI, it has not been proven superior to other forms of physical rehabilitation, and its effects on lower extremity motor score have been mixed [17,46–51]. Treadmill training targets spinal locomotor central pattern generator circuits more strongly than corticospinal circuits [21,22], although cortical circuits may also be modulated [52,53]. The skilled tasks incorporated into MM training, though focused on the upper extremities, likely activated lower extremity neural circuits as well [54]. Regardless, there was a higher degree of participant dropout and variability than expected in this study, leading to changes that did not reach significance for either intervention.

The originally intended primary neurophysiological outcome was tibialis anterior motor evoked potential amplitude (TA MEP). TA MEP depends largely on residual intact corticospinal circuits. However, only two participants had clear TA MEPs at baseline, far fewer than had been anticipated. Therefore, soleus H-reflex facilitation was analyzed as the primary neurophysiological outcome. H-reflex facilitation by subthreshold TMS depends on both direct and indirect connections between cortical and spinal motor neurons [40,41,55]. Facilitation mediated by subthreshold TMS pulses within 20 ms prior to tibial nerve H-reflex pulses (‘short-interval’) likely occurs through direct corticospinal circuits, whereas facilitation mediated by TMS pulses 60–120 ms prior to tibial nerve pulses (‘long-interval’) likely occurs through polysynaptic circuits involving brainstem and spinal pathways [41,44,56].

A trend toward greater H-reflex facilitation was observed in the short-interval window after MM versus TM training (median 2.4% vs -5.9%), consistent with corticospinal facilitation. These data are comparable to our previous results in non-disabled volunteers demonstrating that one session of MM exercise increased short-interval H-reflex facilitation by 6.2% ± 4.0%, whereas one session of TM exercise decreased short-interval facilitation by 1.4% ± 3.8% [16]. Although we did not observe similar changes in long-interval H-reflex facilitation in the current study, those results were much more variable, possibly due to differences in degree of sparing of subcortical pathways among our subjects [40,41].

We know of no other published studies that have reported seated posturography outcomes after rehabilitation interventions in individuals with SCI. MM training did not lead to significantly greater improvement in seated balance performance than TM training did, despite upright postural exercises being incorporated into the MM regimen. Weight-supported treadmill training itself has been shown to improve both gait and clinical balance outcomes [57–59]. We speculate that either upright postural instability during MM training does not transfer efficiently to seated dynamic balance skills, or that simultaneous performance of fine upper extremity tasks interferes with learning or retention of seated dynamic balance skills.

This study has multiple limitations. Participants were more severely impaired than anticipated, making it difficult or impossible to collect data on several of the outcomes. More significantly, fewer than the anticipated number of participants completed the study interventions, limiting the power of this study to detect a difference or to conclusively establish equivalence between the interventions. Including the 6-week washout period, full study participation required at least 30 weeks per subject, which proved burdensome for subjects to maintain, mostly due to transportation issues. In some cases, participants participated in inconsistent numbers of sessions per week, potentially diluting effects of intervention. In others, participants dropped out of the crossover study after completing only a single intervention. This incomplete crossover dataset necessitated a more complicated multilevel statistical analysis, and increased the risk of both Type 1 and Type 2 error. Furthermore, despite attempts to push participants toward the ‘Hard’ level of exertion on the Borg Rating of Perceived Exertion, subjects ranged across a broader range from fairly light (RPE 11) to hard (RPE 15) exertion.

Incorporating lessons from this study should lead to improved yield from future studies–given the prolonged course of each intervention, a parallel-group design, though requiring more subjects, would improve subject retention and simplify statistical analysis. Confirming presence and stability of baseline values across two rather than one screening visit would reduce variability. Finally, we speculate that synergy between cortical and brainstem signaling to the legs may be facilitated by combining balance exercises with concurrent skilled *lower* rather than *upper* extremity exercises. The participants in our study did not generally have the ability to perform skilled lower extremity movements, but perhaps motor imagery may be a mechanism to implement this type of approach [60].

## Conclusion

In participants with chronic incomplete SCI, 48 sessions of a multimodal exercise rehabilitation program incorporating balance exercises with skilled upper extremity exercises showed no benefit compared to 48 sessions of body weight-supported treadmill training. The small number of participants that completed both phases of the crossover intervention limited the power of this study to detect significant effects. Whether a combination of exercises simultaneously stimulating cortical and subcortical circuits may improve rehabilitation in persons with SCI or other neurological conditions remains undetermined.

## Supporting information

S1 File IRB approval letter. (PDF)

S2 File IRB protocol. (PDF)

S3 File Consort checklist. (DOC)

S4 File Subject-level outcome data. (XLSX)

S5 File List of skilled upper extremity tasks. (XLSX)

## References

[1] Sherwood AM, Dimitrijevic MR, McKay WB. Evidence of subclinical brain influence in clinically complete spinal cord injury: discomplete SCI. J Neurol Sci. 1992/07/01. 1992;110(1–2):90–8. 1506875 10.1016/0022-510x(92)90014-c

[2] Kakulas BA. A review of the neuropathology of human spinal cord injury with emphasis on special features. J Spinal Cord Med. 2000/05/29. 1999;22(2):119–24. 10826269 10.1080/10790268.1999.11719557

[3] Ballermann M, Fouad K. Spontaneous locomotor recovery in spinal cord injured rats is accompanied by anatomical plasticity of reticulospinal fibers. Eur J Neurosci. 2006/04/25. 2006;23(8):1988–96. 10.1111/j.1460-9568.2006.04726.x 16630047

[4] Reed WR, Shum-Siu A, Magnuson DSK. Reticulospinal pathways in the ventrolateral funiculus with terminations in the cervical and lumbar enlargements of the adult rat spinal cord. Neuroscience. 2008 1 24;151(2):505–17. 10.1016/j.neuroscience.2007.10.025 18065156 PMC2829753

[5] Sasaki S, Isa T, Pettersson LG, Alstermark B, Naito K, Yoshimura K, et al Dexterous finger movements in primate without monosynaptic corticomotoneuronal excitation. J Neurophysiol. 2004;92(5):3142–7. 10.1152/jn.00342.2004 15175371

[6] Riddle CN, Edgley SA, Baker SN. Direct and indirect connections with upper limb motoneurons from the primate reticulospinal tract. J Neurosci. 2009;29(15):4993–9. 10.1523/JNEUROSCI.3720-08.2009 19369568 PMC2690979

[7] Fregosi M, Contestabile A, Hamadjida A, Rouiller EM. Corticobulbar projections from distinct motor cortical areas to the reticular formation in macaque monkeys. Barbas H, editor. Eur J Neurosci. 2017 6;45(11):1379–95. 10.1111/ejn.13576 28394483

[8] Herbert WJ, Powell K, Buford JA. Evidence for a role of the reticulospinal system in recovery of skilled reaching after cortical stroke: initial results from a model of ischemic cortical injury. Exp Brain Res. 2015;233(11):3231–51. 10.1007/s00221-015-4390-x 26231990

[9] Lemon RN. Descending pathways in motor control. Annu Rev Neurosci. 2008;31:195–218. 10.1146/annurev.neuro.31.060407.125547 18558853

[10] Bareyre FM, Kerschensteiner M, Raineteau O, Mettenleiter TC, Weinmann O, Schwab ME. The injured spinal cord spontaneously forms a new intraspinal circuit in adult rats. Nat Neurosci. 2004;7(3):269–77. 10.1038/nn1195 14966523

[11] Courtine G, Song B, Roy RR, Zhong H, Herrmann JE, Ao Y, et al Recovery of supraspinal control of stepping via indirect propriospinal relay connections after spinal cord injury. Nat Med. 2008;14(1):69–74. 10.1038/nm1682 18157143 PMC2916740

[12] van den Brand R, Heutschi J, Barraud Q, DiGiovanna J, Bartholdi K, Huerlimann M, et al Restoring voluntary control of locomotion after paralyzing spinal cord injury. Science (80-). 2012 6 1;336(6085):1182–5. 22654062 10.1126/science.1217416

[13] Filli L, Engmann AK, Zörner B, Weinmann O, Moraitis T, Gullo M, et al Bridging the gap: a reticulo-propriospinal detour bypassing an incomplete spinal cord injury. J Neurosci. 2014 10 1;34(40):13399–410. 10.1523/JNEUROSCI.0701-14.2014 25274818 PMC6608315

[14] Harel NY, Song KH, Tang X, Strittmatter SM. Nogo Receptor Deletion and Multimodal Exercise Improve Distinct Aspects of Recovery in Cervical Spinal Cord Injury. J Neurotrauma. 2010;27(11):2055–66. 10.1089/neu.2010.1491 20809785 PMC2978056

[15] Harel NY, Yigitkanli K, Fu Y, Cafferty WBJ, Strittmatter SM. Multimodal exercises simultaneously stimulating cortical and brainstem pathways after unilateral corticospinal lesion. Brain Res. 2013 11 13;1538:17–25. 10.1016/j.brainres.2013.07.012 24055330 PMC3873870

[16] Harel NY, Martinez SA, Knezevic S, Asselin PK, Spungen AM. Acute changes in soleus H-reflex facilitation and central motor conduction after targeted physical exercises. J Electromyogr Kinesiol. 2015 3 2;25(3):438–43. 10.1016/j.jelekin.2015.02.009 25771437

[17] Dobkin B, Apple D, Barbeau H, Basso M, Behrman A, Deforge D, et al Weight-supported treadmill vs over-ground training for walking after acute incomplete SCI. Neurology. 2006;66(4):484–93. 10.1212/01.wnl.0000202600.72018.39 16505299 PMC4102098

[18] Field-Fote EC, Roach KE. Influence of a locomotor training approach on walking speed and distance in people with chronic spinal cord injury: a randomized clinical trial. Phys Ther. 2010/11/06. 2011;91(1):48–60. 10.2522/ptj.20090359 21051593 PMC3017322

[19] Yang JF, Musselman KE, Livingstone D, Brunton K, Hendricks G, Hill D, et al Repetitive Mass Practice or Focused Precise Practice for Retraining Walking After Incomplete Spinal Cord Injury? A Pilot Randomized Clinical Trial. Neurorehabil Neural Repair. 2014 11 8;28(4):314–24. 10.1177/1545968313508473 24213960

[20] Brazg G, Fahey M, Holleran CL, Connolly M, Woodward J, Hennessy PW, et al Effects of Training Intensity on Locomotor Performance in Individuals With Chronic Spinal Cord Injury: A Randomized Crossover Study. Neurorehabil Neural Repair. 2017 10 30;31(10–11):944–54. 10.1177/1545968317731538 29081250 PMC5729047

[21] Hubli M, Dietz V. The physiological basis of neurorehabilitation—locomotor training after spinal cord injury. J Neuroeng Rehabil. 2013 1;10:5 10.1186/1743-0003-10-5 23336934 PMC3584845

[22] Barriere G, Leblond H, Provencher J, Rossignol S. Prominent role of the spinal central pattern generator in the recovery of locomotion after partial spinal cord injuries. J Neurosci. 2008/04/11. 2008;28(15):3976–87. 10.1523/JNEUROSCI.5692-07.2008 18400897 PMC6670475

[23] Perez MA, Lungholt BK, Nyborg K, Nielsen JB. Motor skill training induces changes in the excitability of the leg cortical area in healthy humans. Exp Brain Res. 2004/11/19. 2004;159(2):197–205. 10.1007/s00221-004-1947-5 15549279

[24] Jensen JL, Marstrand PC, Nielsen JB. Motor skill training and strength training are associated with different plastic changes in the central nervous system. J Appl Physiol. 2005/05/14. 2005;99(4):1558–68. 10.1152/japplphysiol.01408.2004 15890749

[25] Borg G. Psychophysical scaling with applications in physical work and the perception of exertion. Scand J Work Env Heal. 1990/01/01. 1990;16 Suppl 1:55–8. 10.5271/sjweh.1815 2345867

[26] Bernhardt KA, Beck LA, Lamb JL, Kaufman KR, Amin S, Wuermser L-A. Weight bearing through lower limbs in a standing frame with and without arm support and low-magnitude whole-body vibration in men and women with complete motor paraplegia. Am J Phys Med Rehabil. 2012 4;91(4):300–8. 10.1097/PHM.0b013e31824aab03 22407161 PMC3913065

[27] Adams MM, Ditor DS, Tarnopolsky MA, Phillips SM, McCartney N, Hicks AL. The effect of body weight-supported treadmill training on muscle morphology in an individual with chronic, motor-complete spinal cord injury: A case study. J Spinal Cord Med. 2006;29(2):167–71. 16739562 10.1080/10790268.2006.11753860 PMC1864805

[28] Knikou M. Functional reorganization of soleus H-reflex modulation during stepping after robotic-assisted step training in people with complete and incomplete spinal cord injury. Exp Brain Res. 2013 5 25; 10.1007/s00221-013-3560-y 23708757

[29] Lemon RN, Griffiths J. Comparing the function of the corticospinal system in different species: organizational differences for motor specialization? Muscle Nerve. 2005/04/05. 2005;32(3):261–79. 10.1002/mus.20333 15806550

[30] Brogardh C, Johansson FW, Nygren F, Sjolund BH. Mode of hand training determines cortical reorganisation: a randomized controlled study in healthy adults. J Rehabil Med. 2010/09/03. 2010;42(8):789–94. 10.2340/16501977-0588 20809062

[31] Kirshblum S, Waring W. Updates for the International Standards for Neurological Classification of Spinal Cord Injury. Phys Med Rehabil Clin N Am. 2014 8;25(3):505–17. 10.1016/j.pmr.2014.04.001 25064785

[32] Berg KO, Wood-Dauphinee SL, Williams JI, Maki B. Measuring balance in the elderly: validation of an instrument. Can J Public Heal. 1992/07/01. 1992;83 Suppl 2:S7–11. 1468055

[33] Harel NY, Asselin PK, Fineberg DB, Pisano TJ, Bauman WA, Spungen AM. Adaptation of Computerized Posturography to Assess Seated Balance in Persons with Spinal Cord Injury. J Spinal Cord Med. 2013;36(2):127–133. 10.1179/2045772312Y.0000000053 23809527 PMC3595960

[34] Bohannon RW, Smith MB. Interrater reliability of a modified Ashworth scale of muscle spasticity. Phys Ther. 1987 2;67(2):206–7. 3809245 10.1093/ptj/67.2.206

[35] Adams MM, Ginis KAM, Hicks AL. The spinal cord injury spasticity evaluation tool: development and evaluation. Arch Phys Med Rehabil. 2007 9;88(9):1185–92. 10.1016/j.apmr.2007.06.012 17826466

[36] Melzack R. The short-form McGill Pain Questionnaire. Pain. 1987/08/01. 1987;30(2):191–7. 3670870 10.1016/0304-3959(87)91074-8

[37] Yukawa Y, Kato F, Ito K, Horie Y, Nakashima H, Masaaki M, et al “Ten second step test” as a new quantifiable parameter of cervical myelopathy. Spine (Phila Pa 1976). 2009/01/08. 2009 1 1; 34(1):82–6. 19127165 10.1097/BRS.0b013e31818e2b19

[38] Rossi S, Hallett M, Rossini PM, Pascual-Leone A. Safety, ethical considerations, and application guidelines for the use of transcranial magnetic stimulation in clinical practice and research. Clin Neurophysiol. 2009 12;120(12):2008–39. 10.1016/j.clinph.2009.08.016 19833552 PMC3260536

[39] Floyd AG, Yu QP, Piboolnurak P, Tang MX, Fang Y, Smith WA, et al Transcranial magnetic stimulation in ALS: utility of central motor conduction tests. Neurology. 2009;72(6):498–504. 10.1212/01.wnl.0000341933.97883.a4 19204259 PMC2677511

[40] Serranova T, Valls-Sole J, Munoz E, Genis D, Jech R, Seeman P. Abnormal corticospinal tract modulation of the soleus H reflex in patients with pure spastic paraparesis. Neurosci Lett. 2008;437(1):15–9. 10.1016/j.neulet.2008.03.068 18434014

[41] Wolfe DL, Hayes KC, Potter PJ, Delaney GA. Conditioning lower limb H-reflexes by transcranial magnetic stimulation of motor cortex reveals preserved innervation in SCI patients. J Neurotrauma. 1996 6;13(6):281–91. 10.1089/neu.1996.13.281 8835796

[42] Jankowska E, Hammar I, Slawinska U, Maleszak K, Edgley SA. Neuronal basis of crossed actions from the reticular formation on feline hindlimb motoneurons. J Neurosci. 2003;23:1867–78. 12629191 10.1523/JNEUROSCI.23-05-01867.2003 PMC1890022

[43] Sindhurakar A, Butensky SD, Meyers E, Santos J, Bethea T, Khalili A, et al An Automated Test of Rat Forelimb Supination Quantifies Motor Function Loss and Recovery After Corticospinal Injury. Neurorehabil Neural Repair. 2017;31(2):122–32. 10.1177/1545968316662528 27530125 PMC5243185

[44] Iles JF, Ali AS, Savic G. Vestibular-evoked muscle responses in patients with spinal cord injury. Brain. 2004;127(Pt 7):1584–92. 10.1093/brain/awh173 15128616

[45] Lalonde R, Strazielle C. Brain regions and genes affecting postural control. Prog Neurobiol. 2007/01/16. 2007;81(1):45–60. 10.1016/j.pneurobio.2006.11.005 17222959

[46] Behrman AL, Harkema SJ. Locomotor training after human spinal cord injury: a series of case studies. Phys Ther. 2000 7;80(7):688–700. 10869131

[47] Wirz M, Zemon DH, Rupp R, Scheel A, Colombo G, Dietz V, et al Effectiveness of automated locomotor training in patients with chronic incomplete spinal cord injury: a multicenter trial. Arch Phys Med Rehabil. 2005/04/14. 2005;86(4):672–80. 10.1016/j.apmr.2004.08.004 15827916

[48] Forrest GF, Sisto SA, Barbeau H, Kirshblum SC, Wilen J, Bond Q, et al Neuromotor and musculoskeletal responses to locomotor training for an individual with chronic motor complete AIS-B spinal cord injury. J Spinal Cord Med. 2008 1;31(5):509–21. 19086708 10.1080/10790268.2008.11753646 PMC2607123

[49] Alexeeva N, Sames C, Jacobs PL, Hobday L, Distasio MM, Mitchell SA, et al Comparison of training methods to improve walking in persons with chronic spinal cord injury: a randomized clinical trial. J Spinal Cord Med. 2011 1;34(4):362–79. 10.1179/2045772311Y.0000000018 21903010 PMC3152808

[50] Morawietz C, Moffat F, M C., M F. Effects of Locomotor Training After Incomplete Spinal Cord Injury: A Systematic Review. Arch Phys Med Rehabil. 2013;94(11):2297–308. 10.1016/j.apmr.2013.06.023 23850614

[51] Labruyère R, van Hedel HJ. Strength training versus robot-assisted gait training after incomplete spinal cord injury: a randomized pilot study in patients depending on walking assistance. J Neuroeng Rehabil. 2014 1 9;11(1):4. 24401143 10.1186/1743-0003-11-4 PMC3905290

[52] Thomas SL, Gorassini MA. Increases in corticospinal tract function by treadmill training after incomplete spinal cord injury. J Neurophysiol. 2005/07/08. 2005;94(4):2844–55. 10.1152/jn.00532.2005 16000519

[53] Winchester P, McColl R, Querry R, Foreman N, Mosby J, Tansey K, et al Changes in supraspinal activation patterns following robotic locomotor therapy in motor-incomplete spinal cord injury. Neurorehabil Neural Repair. 2005 12;19(4):313–24. 10.1177/1545968305281515 16263963

[54] Ferris DP, Huang HJ, Kao PC. Moving the arms to activate the legs. Exerc Sport Sci Rev. 2006;34(3):113–20. 16829738 10.1249/00003677-200607000-00005

[55] Benito Penalva J, Opisso E, Medina J, Corrons M, Kumru H, Vidal J, et al H reflex modulation by transcranial magnetic stimulation in spinal cord injury subjects after gait training with electromechanical systems. Spinal Cord. 2010;48(5):400–6. 10.1038/sc.2009.151 19935755

[56] Sand Sivertsen M, Glover JC, Perreault M-C. Organization of pontine reticulospinal inputs to motoneurons controlling axial and limb muscles in the neonatal mouse. J Neurophysiol. 2014 6 18; 10.1152/jn.00820.2013 PMC4631545 24944221

[57] Behrman AL, Ardolino E, Vanhiel LR, Kern M, Atkinson D, Lorenz DJ, et al Assessment of functional improvement without compensation reduces variability of outcome measures after human spinal cord injury. Arch Phys Med Rehabil. 2012 9;93(9):1518–29. 10.1016/j.apmr.2011.04.027 22920449

[58] Harkema SJ, Schmidt-Read M, Lorenz DJ, Edgerton VR, Behrman AL. Balance and ambulation improvements in individuals with chronic incomplete spinal cord injury using locomotor training-based rehabilitation. Arch Phys Med Rehabil. 2012 9;93(9):1508–17. 10.1016/j.apmr.2011.01.024 21777905

[59] Navarrete-Opazo A, Alcayaga JJ, Sepúlveda O, Varas G. Intermittent Hypoxia and Locomotor Training Enhances Dynamic but Not Standing Balance in Patients With Incomplete Spinal Cord Injury. Arch Phys Med Rehabil. 2017 3;98(3):415–24. 10.1016/j.apmr.2016.09.114 27702556

[60] Taube W, Mouthon M, Leukel C, Hoogewoud H-M, Annoni J-M, Keller M. Brain activity during observation and motor imagery of different balance tasks: An fMRI study. Cortex. 2015 3 27;64:102–14. 10.1016/j.cortex.2014.09.022 25461711

