# Supplementary material for: Multimodal cortical and subcortical exercise compared with treadmill training for spinal cord injury
Source: PLoS One. 2018 Aug 9;13(8):e0202130. doi: 10.1371/journal.pone.0202130 (PMC6084979; doi:10.1371/journal.pone.0202130)
Supplement: S1 File — (PDF) [file pone.0202130.s001.pdf]

Subcommittee Research Safety
Bronx VA Medical Center
Research & Development Program (151)
130 West Kingsbridge Road • Bronx, NY 10468 • 718-741-4228 • Fax: 718-741-3937

APPROVAL - Continuing Review

Date: April 19, 2017
From: Rita De Gasperi, Ph.D., Chairperson
Investigator: Noam Harel, M.D.
Protocol: A Hebbian Approach to Regaining Control of Spared Circuits After Spinal Cord Injury
ID: 01407 Prom#: N/A Protocol#: SPU-11-077

The following items were reviewed and approved at the 04/12/2017 meeting:

- Abstract (Protocol Summary) (03/13/2017)
- Annual Adverse Event Report Form (03/13/2017)
- Annual Enrollment Form (03/13/2017)
- Budget Page (03/13/2017)
- Consent Form (03/13/2017)
- Continuing Review (03/13/2017)
- Lay Research Summary (03/13/2017)
- Personnel Record (03/13/2017)
- PI Annual Research Protocol Safety Update (03/13/2017)
- Progress Report (03/13/2017)
- Research Financial Conflict of Interest Statement (03/13/2017)
- Research Protocol Safety Survey-form 10-0398 (03/13/2017)
- Research Data Inventory Form (03/13/2017)
- Signature Page(s) (03/13/2017)
- Training (03/13/2017)

There are no biosafety issues associated with this study. Therefore, this protocol is EXEMPT from future annual review by the Subcommittee Research Safety(SRS).

****Note: If any changes are made to the protocol, an updated research biosafety survey form[10-0398] must be included and submitted for review by the SRS.****

The following other committee reviews are scheduled:
Institutional Review Board [03/01/2018]

Approval by each of the following is required prior to study continuation:
Institutional Review Board [Approval Granted 04/06/2017]
Research & Development Committee
