# Supplementary material for: Multimodal cortical and subcortical exercise compared with treadmill training for spinal cord injury
Source: PLoS One. 2018 Aug 9;13(8):e0202130. doi: 10.1371/journal.pone.0202130 (PMC6084979; doi:10.1371/journal.pone.0202130)
Supplement: S2 File — (PDF) [file pone.0202130.s002.pdf]

PART II
GUIDE TO RESEARCH PROPOSAL

Please use the following guidelines to describe your research protocol on separate sheets, succinctly, but with sufficient information to allow evaluation by an expert consultant in your field. Use the following headings. A Sponsor protocol or grant can be substituted.

1. SPECIFIC AIMS.

To better define the extent of damage and sparing of specific neural circuits after spinal cord injury (SCI). To compare the efficacy of an exercise training regimen simultaneously targeting brainstem and corticospinal circuits to a traditional treadmill training regimen in a population of chronic SCI participants.

Observational Phase: Functional and electrophysiological assessment of residual circuitry in chronic spinal cord injury (SCI)

- **A.** Functional Assessments: sensory and lower extremity motor scores on American Spinal Injury Association Impairment Scale (AIS); Berg Balance Scale; modified Ashworth Scale; Spinal Cord Assessment Tool for Spasticity (SCATS); Walking Index for Spinal Cord Injury II (WISCI II); 10-meter walk test (ambulatory Participants only); steps taken on the 10-second Step Test (AIS C/D Participants only); grasp-and-release test (cervical-level participants only); pinch dynamometry (cervical-level participants only)Spinal Cord Injury Spasticity Evaluation Tool (SCI-SET); WHOQOL-BREF and SCIQoL quality of life questionnaires; and the McGill Pain Questionnaire (short form).
- **B.** Electrophysiological Assessments: Motor evoked potentials; H-reflex testing; seated and standing dynamic posturography; and quality of coordinated muscle activation assessed by surface electromyography.

Interventional Phase: Targeted exercise training for motor-incomplete thoracic SCI

- **A.** Interventions: Participants with motor-incomplete SCI (AIS grade C or D or volitional strength of at least 1/5 in two or more key lower extremity muscles, AND volitional strength of at least 3/5 in shoulder and elbow muscles) or some participants with electromyographic evidence for residual motor circuits will undergo two different exercise training regimens in random order: conventional locomotor treadmill training and multimodal training combining balance training with skilled hand exercises. A harness and two clinicians will be used for support and safety.
    1. LOCOMOTOR: harness-supported treadmill training.
    2. MULTIMODAL: staged balance training (to stimulate brainstem postural circuits) plus skilled hand exercises (to simultaneously stimulate corticospinal circuits);
- **B.** Schedule: Training sessions of 30 minutes will occur 3-4 times per week for a total of 48 sessions per intervention. The functional and electrophysiological parameters described in Phase 1 will be assessed at the beginning and end of each training period; and 6 weeks after completion of all training.
- **C.** Primary Outcomes: Change in tibialis anterior motor evoked potential amplitude; change in lower extremity motor score.
- **D.** Secondary Outcomes: Change in AIS sensory score; change in Berg Balance Scale score; change in number of steps taken during Ten Second Step Test; change in gait speed and disability; change in leg spasticity (modified Ashworth Scale, SCATS, and SCI-SET); change in grasp-and-release test or pinch dynamometry (in cervical-level participants); change in pain (McGill Pain Questionnaire); change in quality of life (WHOQOL-BREF and SCIQoL) change in upper and lower extremity lean tissue mass; change in soleus H-reflex facilitation; change in seated and standing posturography; and change in motor control measured by surface electromyography.

We hypothesize that in motor incomplete SCI participants, combinations of exercises simultaneously stimulating corticospinal and brainstem tracts *above* spinal lesions should lead to greater neural recovery *below* lesions than traditional treadmill training.

Please note: The training protocols described in this proposal involve interventions and safety mechanisms that are similar to other clinical and research protocols already in extensive use both in our SCI rehabilitation service and our SCI Center of Excellence. Therefore, the risks of the training intervention are not expected to be larger than those of standard SCI rehabilitation or our other research protocols such as the Lokomat and ReWalk protocols.

Diagnostically, this protocol introduces several assessment techniques that are new to our center – three of these techniques, computerized posturography, surface electromyography, and H-reflex facilitation testing, do not pose significant new risks. One of these techniques, motor evoked potentials, involves transcranial magnetic stimulation (TMS) – the risks of this procedure, and the extensive safeguards we will use, will be extensively discussed.

2. BRIEF REVIEW OF RESULTS OF OTHERS AND CURRENT STATE OF KNOWLEDGE

The spared fibers of spinal cord injury

Traumatic spinal cord injury (SCI) only rarely leads to complete cord transection – most injuries occur through external trauma to the spinal column, leading to varying degrees of cord contusion (NSCISC 2013). Therefore, rather than severing the cord, most injuries leave a portion of the cord's nervous tissue intact (Hayes & Kakulas 1997; Kakulas 1987). Even in patients who cannot consciously sense or move their body below a lesion, there is often some degree of motor, sensory, and/or autonomic circuit sparing (McKay et al. 2004; Sherwood et al. 1992).

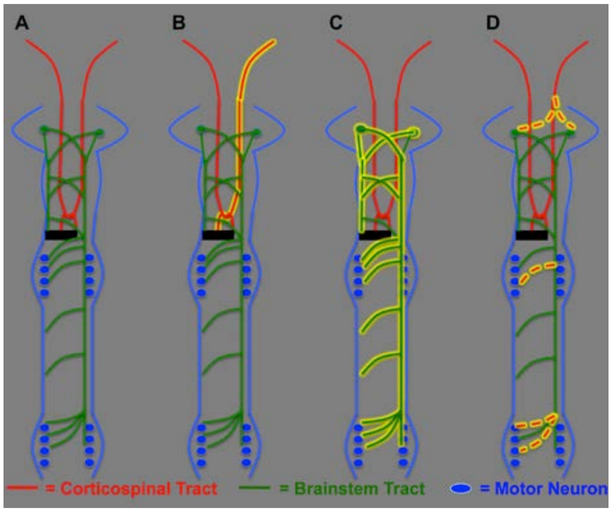

Figure 1 – Schematic of corticospinal (red) and brainstem tracts (green) after anatomically incomplete cervical spinal cord injury (A). Note that only brainstem fibers survive below the lesion. Repetitive simultaneous firing of CST and brainstem tracts (B-C) leads to strengthening of alternative connections between CST fibers and brainstem neurons. In this fashion, a detour pathway reforms between the cortex and motor neurons on the previously denervated side of the cord (D).

Spared circuits represent a potential pathway for recovery – therefore, it is crucial to characterize their identity in individual patients. Due to their anatomically diffuse distribution, fibers of brainstem and spinal tracts (for example, the reticulospinal tract) are much more likely to survive cord damage than those of cortically-originating tracts such as the corticospinal tract (CST) (Jankowska & Edgley 2006; Nathan et al. 1996).

If we were better able to detect and characterize spared neural pathways, clinicians could give patients not only a more accurate prognosis, but perhaps better treatment – that is, interventions more specifically targeted toward spared circuits could improve rehabilitation outcomes. This proposal presents the rationale, preliminary data, and research design intended to achieve these goals.

Recovery through Rerouting, not Regeneration

To re-establish conscious control over brainstem and spinal circuits, treatments need to connect brain centers that initiate voluntary movement with the spinal centers that execute that movement (**Figure 1**). These types of rerouted detour connections mediate functional recovery in animal SCI models even without specific treatment (Bareyre et al. 2004; Courtine et al. 2008). We aim to improve this process.

Fire together, Wire together

One approach to improving connectivity and function after CNS injury involves increasing activation of the nervous system itself. Activity-inducing treatments include repetitive transcranial magnetic stimulation (TMS), transcranial direct current stimulation (tDCS), and various forms of implanted electrical stimulation (Brus-Ramer et al. 2007; Carmel et al. 2010; Courtine et al. 2009; Harkema et al. 2011; Minassian et al. 2007; Sadowsky & McDonald 2009).

We propose to use a non-invasive method of neural activation – repetitive exercise training – to improve connectivity of the injured spinal cord. Our approach to exercise training for SCI builds on the principle of 'Fire together, Wire together' made famous by Donald O. Hebb (Hebb 1949): When nearby neurons repetitively fire in sequence, they are more likely to form new and/or stronger physiological connections.

The CST sends many collateral fibers toward brainstem nuclei as it descends toward the spinal cord (Jankowska & Edgley 2006; Matsuyama et al. 2004). We plan to strengthen these collateral connections by using targeted repetitive exercises that stimulate nerves from cortical and brainstem pathways simultaneously (**Figure 1**). We have begun demonstrating the feasibility of this approach in mouse models of central nervous system injury (Harel et al. 2010; Harel et al. 2013). We now intend to translate this approach to human patients.

Data from other studies in human Participants

Balance training on an unstable surface improves postural control in chronic thoracic SCI patients (Kim et al. 2010) – In a small study that shares similarities with our proposed study, Kim et al. tested the effects of balance training in a chronic thoracic SCI population. Seven participants with motor-complete (AIS A or B) injury were subjected to progressively more difficult balance exercises over the course of 20 sessions. Balance training resulted in increased reaching distance and decreased postural sway relative to patients receiving 'conventional' therapy (**Figure 2**). This demonstrates the effectiveness of a staged balance training program such as the one we propose to use in our participants, as well as the feasibility of tracking outcomes using computerized posturography. As detailed in the Research Design, we plan on utilizing a related balance training protocol with a greater number of participants, more finely differentiated control treatment, and more sensitive outcome measures.

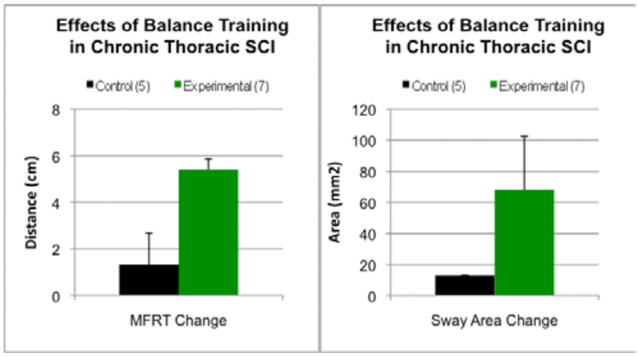

Figure 2 – Balance training on an unstable surface improves reach and postural sway in chronic thoracic SCI patients. **(A)** Increased distance achieved on Modified Functional Reach Test (MFRT). **(B)** Larger reduction in sway area determined by computerized posturography. Adapted from Kim et al., 2010.

Detailed surface electromyography (EMG) recordings sensitively track improved patterns of motor control over time in SCI patients (McKay et al. 2010) – McKay and colleagues have repeatedly shown the value of using surface EMG to glean information about residual neural connections and motor control after SCI. In this recent demonstration, they performed extensive EMG evaluation on acutely injured patients 1 to 11 days post-SCI, then performed repeated assessments over the next 3-17 months. They were able to electrographically track progressive improvement in overall coordination and control of major

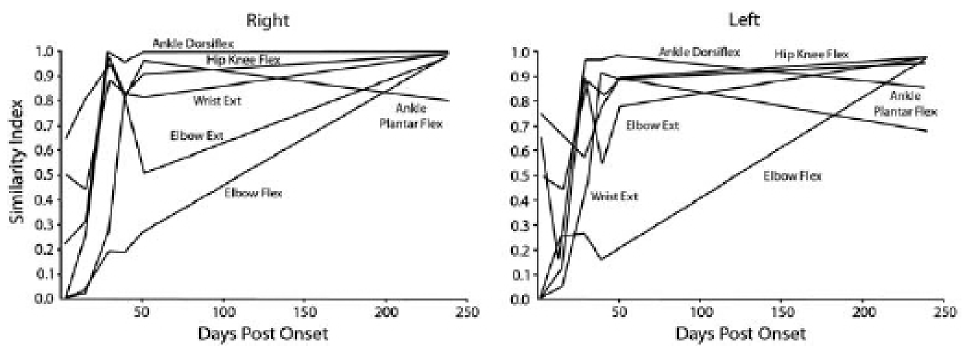

Figure 3 – Progressive improvement in electrographic motor control over various muscle movements in an individual SCI patient over time. Adapted from McKay et al., 2010.

muscle groups over time (**Figure 3**). Importantly, this type of analysis tracks changes in *motor control* more sensitively than clinical examination or other types of assessments of *muscle movement*. We intend to continue developing this tool as a powerful biomarker for underlying changes in neural circuitry in response to various treatments.

3. PROCEDURES, METHODS AND EXPERIMENTAL DESIGN.

Participant Inclusion Criteria:

Phase 1 Functional and electrophysiological assessment of residual circuitry

1. Age between 21 and 65 years;
2. Chronic thoracic SCI (levels C2-T12; more than 12 months since injury);
3. AIS grades A, B, C, or D.

Phase 2: Targeted exercise interventions

1. Age between 21 and 65 years;
2. Chronic thoracic SCI (levels C2-T12; more than 12 months since injury);
3. AIS grades C or D or volitional strength of at least 1/5 in two or more key lower extremity muscles; or some participants with electromyographic evidence for residual motor circuits;
4. Strength of at least 3/5 in shoulder and elbow muscles;
5. Able to tolerate standing with support;
6. Morphologically capable of fitting a weight-support harness and robotic treadmill system: measurements of the greater trochanter to lateral epicondyle between 35-47 cm for placement in the thigh cuffs; pelvic width >50 cm; thigh circumference above the knee <57 cm; and body mass <135 kg.

Participant Exclusion Criteria:

1. Unsuitable cognitive capacity as judged by the study physician;
2. Diagnosis of neurological injury other than SCI, such as stroke, traumatic brain injury; or other neurological condition that the study physician considers to be exclusionary;
3. Multiple spinal cord lesions;
4. History of frequent autonomic dysreflexia;
5. History of seizures;
6. Use of medications that significantly lower seizure threshold, such as anti-psychotics, tricyclic antidepressants, amphetamines, and bupropion;
7. History of implanted brain/spine/nerve stimulators, aneurysm clips, or cardiac pacemaker/defibrillator;
8. Deep vein thromboses in lower extremities of less than 6 months duration;
9. Pregnancy;
10. (Interventional phase only): Lack of detectable tibialis anterior MEP at baseline even with active facilitation;
11. (Interventional phase only): Inability to maintain supported upright posture without symptoms of orthostatic hypotension (light-headedness, pre-syncope);
12. (Interventional phase only): Active pressure ulcers greater than stage 1 severity on the foot, ankle, knee and/or pelvic girdle;
13. (Interventional phase only): Bone mineral density of the hip (proximal femur) T-score <3.5 SD from age- and gender-matched normative data;
14. (Interventional phase only): Any spasticity, contractures, or heterotopic ossification that result in inadequate range of motion of the shoulder, elbow, wrist, fingers, hip, knee, or ankle joints in the judgment of the study physician;
15. (Interventional phase only): Inability to cooperate with instructions or unwilling to commit to training sessions for three-four days per week over roughly 9 months;
16. (Interventional phase only): A diagnosis of coronary artery disease that precludes moderate to intense exercise.

Recruitment:

Recruitment will be accomplished by pre-existing relationships, physician referrals, and IRB-approved advertisements. Veterans with SCI who have an ongoing relationship with the Center of Excellence for the Medical Consequences of SCI, such as those who attend the pulmonary, endocrine, cardiovascular, and gastroenterology clinics, will be informed about the study. Physicians at the James J. Peters VA Medical Center will be informed of the goals and aims and the inclusion/exclusion criteria for this study. We will not contact any patients unless referring physicians provide us with assurance that the patient would be interested in receiving further information about this study.

Eligibility Screening:

Persons with SCI interested in participating will be assigned a number beginning at 001. No PHI will be collected at that time. To determine eligibility, interested participants will be asked the “yes” or “no” questions listed below. Persons who answer “no” to any of the following questions will not be eligible for the study:

- Do you have a cervical or thoracic spinal cord injury (SCI)?
- Did your SCI occur greater than 12 months ago?
- Are you between the ages of 21 to 65 years?
- Are you legally able to sign for your own consent?

If the potential participant answers “yes” to all questions, then the informed consent process will continue by inviting the participant for an in-person interview.

Enrollment:

At the in-person interview, the study will be explained by one of the study team members. All study team members will be trained to obtain consent. The study will be explained in its entirety. The study team member will explain that the training regimens are being studied for investigational purposes. Along with explaining each of the testing and evaluation procedures, the study team member will explain every possible risk that the participant may encounter. No benefits will be guaranteed.

Included in this explanation will be the necessity for the investigative team to determine study eligibility by “screening” – screening will include a review of the participant’s medical record, a DXA scan, and a physical examination by the study physician. The time commitment that is needed to be a part of this study will also be explained – multiple visits per week for a period of approximately four months. The potential participant will be told that there is a possibility that he or she will not be eligible if any of the exclusion criteria are found to be true (i.e. a screening failure). It will also be explained to the participant that if he or she misses more than 25% of the study visits, then the investigators have the option to remove that person from the study.

Potential participants will be encouraged to ask questions throughout the process. Potential participants will be informed of their right to withdraw at any time and that choosing to not participate will not infringe on any of their regular VA benefits or medical care. Once he/she has no further questions and the study team is confident that the potential participant fully understands the protocol and its risks, then the participant will be asked whether he or she is willing to sign the ICF.

Baseline testing (to be conducted over two visits):

Once a participant has provided signed informed consent, a detailed review of the participant’s history and medical record will be conducted. A physical exam will be performed, including the Modified Ashworth Scale, the Spinal Cord Assessment Tool for Spasticity (SCATS) (Benz et al. 2005), and the American Spinal Injury Association (ASIA) Impairment Scale (AIS). Further clinical assessments will include the Berg Balance Scale; the Walking Index for Spinal Cord Injury II; the 10-meter walk test; the 10-second seated step test; a modified grasp-and-release test (Wuolle et al., 1994); pinch dynamometry; the Spinal Cord Injury Spasticity Evaluation Tool (SCI-SET) (Adams et al. 2007); the WHOQOL-BREF questionnaire (World Health Organization 1998); SCI-QoL questionnaire (Tulsky et al. 2011); and the

McGill Pain Questionnaire (short form). Depending on the severity of paraparesis, not all Participants will be able to perform the step or walk tests. As such, no baseline walking data will be collected for these outcomes in these participants. To ensure tolerance of upright position, heart rate and blood pressure will be measured for 10 minutes while the Participant is in an upright position supported using an overhead harness or standing frame. A full-body DXA scan will be performed. To be eligible for Phase 2 of the study, results from the DXA of the proximal femur must not have a T-score lower than 3.5SD below age/gender-matched controls. Seated and standing computerized posturography, motor evoked potentials, multiple tests of soleus H-reflex facilitation, and surface electromyography will also be assessed. At this point, the study evaluation is complete for participants with AIS A/B impairment. Only participants with AIS C/D injury or volitional strength of at least 1/5 in two or more key lower extremity muscles or some participants with electromyographic evidence for residual motor circuits will be eligible for the interventional component of our study. If any exclusion criteria are met, or if the participant decides at any time not to continue, he or she will not be further studied.

Randomization:

Participants with motor incomplete SCI will receive the two training interventions in randomized order, with randomization occurring in blocks of 6 subjects.

**Berg Balance Scale: Sitting with Back Unsupported**
**0** Unable to sit for at least 10 seconds
**1** Able to sit 10 seconds
**2** Able to sit 30 seconds
**3** Able to sit 2 minutes under supervision
**4** Able to sit safely and securely for 2 minutes

Table 1 –Berg Balance Scale: Section on sitting with back unsupported.

Training period (48 sessions per intervention separated by a 6-week washout):

Participants will undergo training with 30-minute sessions during 3-4 visits per week. Participants will have the option of performing up to two training sessions per visit to our center, with a break of at least 45 minutes between sessions. The two training paradigms will consist of 1) LOCOMOTOR training and 2) MULTIMODAL training. Training paradigms are described in detail in the next section.

Post-training evaluation:

All the measures evaluated at the baseline visits will be repeated at the beginning and end of each training period. Evaluation will take place over one or two sessions within one week of the completion of each intervention.

6-Week Follow-up evaluation:

All the measures evaluated at the baseline visits will be repeated at six weeks after completion of the second intervention.

Primary outcomes:

1. Change from baseline in motor evoked potential amplitude in the tibialis anterior muscle; change from baseline in AIS lower extremity motor score.

Secondary outcomes:

1. Change from baseline in AIS sensory scores.
2. Change from baseline in Berg Balance Scale score.
3. Change in leg spasticity on modified Ashworth Scale, Spinal Cord Assessment Tool for Spasticity, and Spinal Cord Injury Spasticity Evaluation Tool.
4. Change in gait speed on 10-meter Walk Test.
5. Change from baseline in Walking Index for Spinal Cord Injury II (WISCI II) scale.

6. Change from baseline in total number of steps taken by both feet during seated 10-second step test.
7. Change from baseline in total number of successful grasp-and-release trials taken by each hand (for cervical-level participants only).
8. Change from baseline in pinch dynamometry strength (in cervical-level participants only).
9. Change in quality of life as determined by WHOQOL-BREF and SCIQoL questionnaires.
10. Change in subjective pain as determined by McGill Pain Questionnaire (short form).
11. Change in percentage of upper and lower extremity lean tissue mass.
12. Change from baseline in endpoint excursion and directional control parameters achieved during seated Limits of Stability testing.
13. Change from baseline in soleus H-reflex facilitation and inhibition using appropriately timed physical stimuli, subthreshold transcranial magnetic stimulation, peroneal and tibial nerve stimulation.
14. Change from baseline in the Voluntary Response Index (VRI) for key leg muscles as detected by surface electromyography(Lee et al. 2004).

Research Design:
This protocol will investigate the efficacy of combining fine-motor exercises with balance exercises in the rehabilitation of chronic thoracic spinal cord injury patients. There are two phases: an observational phase for participants with either motor complete or incomplete SCI; and an interventional phase for participants with motor incomplete SCI. The observational phase will characterize the residual function of specific neural circuits. The interventional phase will employ a single-group, single-blinded, pre-post-intervention crossover research design. We plan to enroll 51 participants for the observational phase. 24 participants with motor incomplete SCI will continue with the interventional phase, lasting approximately 9 months. The protocol will take 3 years to complete. We hypothesize that participants will demonstrate improved neural connectivity when undergoing multimodal training compared to locomotor training.

Statistical Analysis:
For the observational phase, exploratory analyses will be undertaken to determine how well scores on the electrographic outcomes (evoked potentials, H-reflex testing, computerized posturography, surface electromyography) correlate with clinical outcomes such as gait disability, gait speed, and AIS motor and sensory scores. Using post hoc regression analysis, we hope to generate a preliminary model to predict anatomical and functional integrity of specific neural tracts based on performance on this battery of tests. This analysis and any resulting predictive model would need further validation with a prospectively studied population. The interventional phase of the study would not depend on the success of obtaining such a predictive model.

For the interventional phase of the study, participants will be randomly assigned to receive either locomotor training followed by multimodal training, or multimodal training followed by locomotor training. The primary outcomes will be change in tibialis anterior motor-evoked potential amplitude and change in lower extremity motor score (LEMS) compared to baseline. All other outcomes are secondary and exploratory.

The most rigorous method of analyzing data from crossover studies involves the use of independent-sample rather than paired-sample tests (Wellek & Blettner 2012). Therefore, outcome measures will be calculated within each subject as the percent change between baseline and completion of each intervention. The percent change across all subjects for each training modality will be compared using independent-sample t-tests or Mann-Whitney U tests depending on the normality of the data distribution. To test for the possibility that the washout period does not fully eliminate carryover effects, the percent change across all subjects for each training period (pre-washout, post-washout) will also be compared using independent sample tests. Statistical significance for all outcomes will be corrected for multiple comparisons using Bonferroni's method.

Power Calculation: Given that our training protocol is novel, we need to make predictions by extrapolating from other types of protocols. This is further hampered by the fact that most SCI

rehabilitation studies involve rather small numbers of participants in often inadequately controlled studies.

The most applicable study upon which to base our power analysis measured MEP amplitudes before and after 60-100 sessions of treadmill training in eight subjects with incomplete thoracic SCI (Thomas & Gorassini 2005). They found a 46±12% increase in maximal MEP amplitude post-training. Additionally, studies in uninjured volunteers demonstrated a 40-70% increase in MEP amplitudes with skilled training exercises but not passive movement or strength training (Jensen et al. 2005; Perez et al. 2004).

Due to these and other factors, we project an improvement of 50% with MULTIMODAL training versus 25% with TREADMILL training. The use of a crossover design will reduce inter-subject variability between the two training modalities compared to a parallel-group design. We conservatively project an estimated variance of 25% for each group – this magnitude of variance is larger than the average variance seen in the MEP studies cited above. Based on these numbers, the calculated power to detect a difference between the two training groups on a two-tailed independent-sample *t*-test or Mann-Whitney test is greater than 80% with 18 subjects, at an $\alpha = 0.05$. We will enroll 24 subjects to account for up to a 25% dropout rate.

	Year 1	Year 2	Year 3	Total N
**Phase 1**	15	18	18	51
**Phase 2**	6	10	8	24

Table 3 Clinical Study Timeline – Participants enrolled per year.

3a. Basic Information (Even if described under "Procedures", list the information requested below where pertinent.)

The study will occur in two phases: An observational phase, in which participants will undergo a series of clinical and neurophysiological tests; and an interventional phase, in which participants with incomplete SCI will undergo standard LOCOMOTOR training and experimental MULTIMODAL training in random order. Training will continue for 48 sessions per intervention, separated by a 6-week washout period. The clinical and neurophysiological tests will be repeated at the end of each intervention.

All participants will undergo baseline testing. Only participants with motor-incomplete SCI (AIS C/D grades or volitional strength of at least 1/5 in two or more key lower extremity muscles AND volitional strength of at least 3/5 in shoulder and elbow muscles) will participate in the exercise training phase as well as the post-training and follow-up assessment testing.

The following procedures will be used:

Clinical Tests

- Physical Examination – A standard physical and neurological exam will be performed. This will include seated and upright blood pressure testing.
- American Spinal Injury Association (ASIA) Impairment Scale – Manual assessment of muscle strength in the arms and legs, as well as assessment for sensation in the arms, trunk, legs, and perineal area.
- Modified Ashworth Scale – Leg muscle spasticity will be qualitatively assessed during the clinical exam on a scale of 0 to 4.
- Spinal Cord Assessment Tool for Spasticity (SCATS) – Three components of spasticity in the lower extremity – clonus, extensor spasm, and plantar response – will be timed in response to standardized evocative maneuvers (Benz et al. 2005).

- Berg Balance Scale – Balance and postural stability will be qualitatively assessed in seated, lying, and standing positions. Most patients will not be able to perform the standing portions of the scale. See **Table 1** for details of seated balance section.
- 10-meter walk test (10MWT) – Ambulatory participants will be asked to walk 10 meters at their best pace. The time to cover this distance will be timed with a stopwatch. Assistive devices will be used if necessary – participants using an assistive device at baseline will be retested with the same assistive device at follow-up visits after training.
- Walking Index for Spinal Cord Injury II – Participants will be graded on a scale of 0-20 based on the degree of assistive device support required for walking 10 meters (Ditunno Jr. et al. 2007).
- Seated 10-second step test – Participants with the ability to move their legs will be seated with their hips and knees flexed to 90 degrees. Steps will consist of lifting one foot entirely off the ground and placing it back down again. The average number of steps taken during three 10-second trials with each foot will be recorded.
- Grasp-and-release test – Participants with cervical-level injury in seated position will be asked to grasp a standardized bottle, pick it up, move it approximately 25 cm, then place it down and release. The average number of successful grasp-and-releases taken during three 30-second trials with each hand will be recorded.
- Pinch dynamometry – In participants with cervical-level injury, pinch strength will be recorded using a wireless dynamometer (JTech Freedom Tracker). The average of three maximal pinch strengths in each hand will be recorded.
- McGill Pain Questionnaire (short form) – Chronic neuropathic pain commonly plagues subjects with SCI. This short (less than 5 minutes), validated survey will provide a baseline to track whether any neural plastic changes result in adverse effects on neuropathic pain.
- Spinal Cord Injury Spasticity Evaluation Tool (SCI-SET) - This survey evaluates how spasticity impacts a person's activities of daily living over the course of the previous 7 days. The survey uses a bidirectional Likert scale to detect both negative and positive impacts of spasticity (Adams et al. 2007).
- SCIQoL - Participants will complete a questionnaire regarding physical/medical health, emotional health, physical function, and social participation. A research assistant will conduct the interview. The questionnaire is located on an online platform called Assessment Center. The Assessment Center database is housed at Northwestern University and analyzed by a team at the University of Michigan. No HIPAA identifiers will be collected through the online platform.
- WHOQOL-BREF Questionnaire – This tool assesses general quality of life, and has been internationally validated for use in the SCI population. It is briefer than the computer-adapted SCI-QOL survey and will be used specifically to correlate with findings on the SCI-SET and SCATS (World Health Organization 1998).

Radiographic/Physiological Tests

- Dual Energy X-ray Absorptiometry (DXA). A whole-body DXA scan will be obtained using a Lunar iDXA (GE) located in our COE on 7A-13. Data will include bone densitometry for the spine, femurs, and knees. There is no discomfort associated with this test. Participants will be asked to lie on a semi-padded, fixed table-top for about 30-40 minutes while being scanned. If the participant's scan is set to the highest setting (e,g,. thicker regional mass will require a higher setting for the scanning mode), the following will be the delivered radiation exposure:

Site	Irradiation time (sec)	Estimated skin entrance dose (μGy)	Effective Dose (μSv)
Total Body	739	6	8.62
Dual Femur	212	329	12.3
AP Spine	104	329	6.8

Knee	54	34	No Data

Table 4 – Radiation exposure during dual energy X-ray absorptiometry. Note: Values of effective dose (absorption) for the knee in μSv do not exist at this time, but considering the intensity and the duration of the scan is less than or equal to that of the other scans, the absorbed amount will also be lower (GE Medical systems, Madison WI).

- Motor evoked potentials (MEP) – This technique will be performed using a MagPro R30 system (MagVenture) located in the COE on 7A-13 (FDA 510K approval #K061645). Muscle responses to TMS and the other procedures described below will be recorded using a Viking Select EMG system (Natus) or a Trigno wireless EMG system (Delsys). Inducing MEPs with transcranial magnetic stimulation is a standard technique used to assess the CST's ability to propagate signals to muscles above or below spinal lesions, through direct and/or detour pathways (McKay et al., 2005). A stimulator coil will be positioned appropriately over the motor cortex to evoke action potentials while recording from key muscle groups: first dorsal interosseous (C8); tibialis anterior (L4); and soleus (S1). Recruitment curves at escalating stimulation intensities will be determined, including the percent output required to reach peak MEP amplitude (MEP_{max}) and the threshold required to produce a detectable MEP (MEP_{thresh}), all as described (Thomas and Gorassini, 2005). Note that this use of TMS does ***not*** involve ***repetitive*** TMS. Repetitive TMS is a separate entity utilized to achieve modulation of brain activity. Even though it is considered to be higher-risk than diagnostic/single pulse TMS, repetitive TMS has been FDA-approved for treatment of refractory major depression. This repetitive TMS device (NeuroStar) is in clinical use at the JJPVAMC Department of Psychiatry (FDA 510K approval #K083538).

- Soleus H-reflex facilitation – All electrical stimuli will be performed using the Viking Select system or a Grass S88 dual-output stimulator (Natus) or a DS7A stimulator (Digitimer). This test provides insight on descending supraspinal influence over a spinal reflex located below the injury level. Electrical pulses (1ms duration) will be applied via surface electrodes in the popliteal fossa (over the tibial nerve) to stimulate the H-reflex, as recorded by electrodes over the soleus muscle. A range of pulse intensities will be delivered to determine the threshold and maximum amplitude of the H-reflex (H_{max}), slope of the H-reflex recruitment curve, and maximal direct soleus response amplitude (maximal M-wave or M_{max}). To test TMS-facilitated H-reflex response, TMS intensity will be set at 90% of resting motor threshold (ie, subthreshold stimulus) (or 80% of maximum stimulator output if no active motor evoked potentials are obtainable from the TA). H-reflex stimulation intensity will be set to elicit an H-reflex of 10-20% of M_{max} (Serranova et al. 2008). A series of paired pulses will be delivered every 10 seconds in intervals of 0-120ms apart (TMS before H-stimulus). The paired-pulse time intervals and unconditioned H-stimuli will be varied at random. At each time interval, the amplitude of the TMS-conditioned H-reflex (averaged over 3-5 stimuli) will be compared with the non-conditioned H-reflex amplitude to determine the magnitude of facilitation. For example, if non-conditioned H-reflex amplitude is 0.5mV, whereas H-stimulus preceded 60ms earlier by subthreshold TMS results in a reflex of amplitude 0.9mV, the magnitude of facilitation would be 180% at that time point. This procedure is already IRB-approved (SPU-12-07).

- Soleus H-reflex pre-synaptic inhibition (PSI) – This test measures the magnitude of peroneal nerve-mediated PSI of tibial nerve Ia afferent fibers. The spinal interneurons involved in this circuit are influenced by descending supraspinal connections(Iles 1996). Peroneal nerve stimulation (at 95% of tibialis anterior motor threshold) will be delivered as 3x1 ms pulses at 333 Hz, 21 ms prior to tibial nerve stimulation (Roche et al. 2011). The amplitude of the conditioned H-reflex (averaged over 3-5 stimuli) will be compared with the non-conditioned H-reflex amplitude to determine the magnitude of inhibition.

- Soleus H_{max}/M_{max} – This measure serves as an electrophysiological proxy for lower limb spasticity(Trimble et al. 2001). It will be derived from the standard procedure of stimulating the tibial nerve over a range of intensities. H-reflex amplitudes will be fitted to a sigmoid

function(Klimstra & Zehr 2008). Tibial nerve stimulation intensity will be increased until M_{max} is obtained. Higher ratios of H_{max} to M_{max} correlate with increased spasticity.

- Soleus H-reflex reciprocal inhibition – Reciprocal inhibition is essential for reducing interference between agonist and antagonist muscles(Knikou & Mummidisetty 2011). This circuit is similar to the PSI circuit described above – peroneal nerve stimulation leads to interneuron-mediated inhibition of soleus alpha motor neurons. As with PSI, the interneurons mediating reciprocal inhibition are influenced by descending brainstem and corticospinal pathways(Knikou & Mummidisetty 2011; Morita et al. 2001). Peroneal nerve stimulation (at 95% tibialis anterior motor threshold) will be delivered at varying intervals prior to tibial nerve stimulation to find the interval and magnitude of maximal inhibition (usually less than 5 ms)(Roche et al. 2011). The amplitude of the conditioned H-reflex (averaged over 3-5 stimuli) will be compared with the non-conditioned H-reflex amplitude to determine the magnitude of inhibition.
- Computerized Posturography – Posturography testing will be performed using the Smart Equitest device located in ENT clinic (2C-07). The Smart Equitest device (Neurocom; FDA 510K approval #K851744) has been in clinical use by the Audiology Clinic for the past four years to test patients with balance and vestibular disorders. The device consists of floor pressure plates embedded in a computerized movable platform. Software-controlled protocols test the effects of different types of balance perturbations on the participant's ability to maintain and adjust the center of gravity. Importantly, posturography testing can be performed in either a standing or sitting position. Response times and muscle activity of the legs (either volitional or reflexive) will be recorded. We will focus on the Limits of Stability (LOS) testing paradigm. In the LOS test, participants are asked to shift their center of gravity toward eight different surrounding targets. Key measures are endpoint excursion of the center of gravity, and the directional accuracy of intended movements – the sensors and software calculate each of these measures automatically.
- Brain Motor Control Assessment (BMCA) – Multichannel EMG assessments will be performed using surface (not needle) electrodes connected to a Viking Select system (Natus) or a Trigno wireless system (Delsys) located in 7A-13. Changes in leg muscle activity will be monitored during relaxation, reinforcement maneuvers (e.g. flexing neck against resistance), and voluntary movements. Key muscle EMG amplitudes and time-to-peak values will be determined. In this way, several measures of motor control and neural responsiveness will be analyzed, providing insight into the presence of residual cortical and brainstem circuitry running through spinal lesions.

Training Paradigms

- LOCOMOTOR – Participants will receive body weight-supported treadmill therapy (BWSTT) using the Lokomat apparatus (Hocoma Medical Engineering) located in 7A-13. COE staff members have significant experience training SCI patients on the Lokomat, including transfer in and out of the apparatus. The Lokomat consists of a robotic exoskeleton, a harness weight support system, and a treadmill, allowing the system to assist participants with leg weakness to engage in repetitive upright step cycles as training to improve gait. This system is already in use for both clinical and research purposes at the JJPVAMC and elsewhere (for example, IRB-approved protocol #4843-06-090). Briefly, each session includes: 15-20 minutes of participant set-up, 5-10 minutes of warm-up, 20-30 minutes of Lokomat therapy, and 5-10 minutes for cool down and to remove the participant from the system. During the Lokomat sessions, heart rate and blood pressure are monitored and recorded by the therapist. Work performed is measured and recorded in the Lokomat computer for time, speed, amount of weight bearing, and distance.
- MULTIMODAL – Subjects will undergo simultaneous balance and skilled upper extremity exercises to stimulate brainstem and corticospinal circuits, respectively. The graded balance-training program will use the same harness weight support system as the Lokomat. At all times, a harness will be used for fall prevention and weight support as needed. Clinicians at the

participant's side will provide trunk/pelvic/leg stabilization as necessary. Each stage involves simultaneous balance and skilled hand exercises.

Balance Exercises: Subjects will be given weight support to maintain upright position while the feet are placed on a semi-spherical balance ball (Figure 4). Aside from the weight-support harness, two clinicians will provide manual trunk/pelvic support and leg adjustments as needed. Subjects will be asked to make an effort to keep the balance surface as stable as possible. Weight support will be gradually titrated downwards as tolerated.

Skilled upper extremity exercises: While undergoing balance exercises, subjects will perform various skilled arm or hand manipulations. One such task will involve inserting different-sized coins into slots oriented at several different angles (**Figure 4**). Subjects will perform alternating series of 4 trials with each hand. This task requires fine motor skills to pick up individual coins, as well as 'pre-shaping' the hand to the orientation corresponding to the coin slot. Cervical-level participants will train with tasks that involve proximal arm aiming and forearm supination. These types of activities have been shown to depend on corticospinal motor circuits (Brogardh et al. 2010; Lemon & Griffiths 2005; Pettersson et al. 2007).

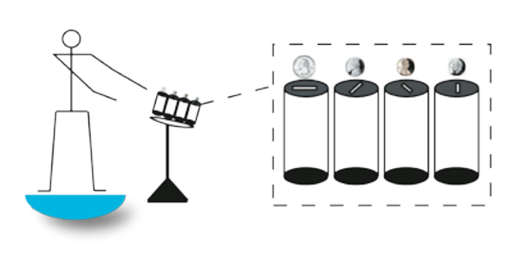

Figure 4 – Multimodal training group. Weight-supported standing balance on unstable surface. Subjects simultaneously perform fine-hand exercises. The coin-sorting task is enlarged at right.

Training Schedule: Exercise sessions will be 30 minutes. Rest periods will be taken as needed, or at least every 10 minutes. Rest periods will consist of lowering the participant to a seated position while in the harness for a period of 1-2 minutes or as needed. Training will occur 3-4 days per week for a total of 48 sessions per intervention. Participants will be allowed a minimum of 12 weeks to complete 48 sessions, but no more than 16 weeks. A six-week washout period will occur between interventions.

Inclusion Criteria:

Phase 1 Functional and electrophysiological assessment of residual circuitry

- Age between 21 and 65 years;
- Chronic thoracic SCI (levels C2-T12; more than 12 months since injury);
- AIS grades A, B, C, or D.

Phase 2: Targeted exercise interventions

- Age between 21 and 65 years;
- Chronic thoracic SCI (levels C2-T12; more than 12 months since injury);
- AIS grades C or D, or volitional strength of at least 1/5 in two or more key lower extremity muscles or some participants with electromyographic evidence for residual motor circuits;
- Strength of at least 3/5 in shoulder and elbow muscles;
- Able to tolerate standing with support;
- Morphologically capable of fitting a weight-support harness and robotic treadmill system: measurements of the greater trochanter to lateral epicondyle between 35 to 47 cm for placement in the thigh adjustments; pelvis width <50cm; thigh circumference above the knee <57cm; and a body mass <135 kg.

Exclusion criteria:

- Unsuitable cognitive capacity as judged by the study physician;

- Diagnosis of neurological injury other than SCI, such as stroke, traumatic brain injury, or other neurological condition that the study physician considers to be exclusionary;
- Multiple spinal cord lesions;
- History of frequent autonomic dysreflexia;
- History of seizures;
- Use of medications that significantly lower seizure threshold, such as anti-psychotics, tricyclic antidepressants, and bupropion;
- History of implanted brain/spine/nerve stimulators, aneurysm clips, or cardiac pacemaker/defibrillator;
- Deep vein thrombosis in lower extremities of less than 6 months duration;
- Pregnancy;
- (Interventional phase only): Lack of detectable tibialis anterior MEP at baseline even with active facilitation;
- (Interventional phase only): Pressure ulcers greater than stage 1 severity on the foot, ankle, knee and/or pelvic girdle;
- (Interventional phase only): Bone mineral density of the hip (proximal femur) T-score <3.5 SD from age- and gender-matched normative data;
- (Interventional phase only): Any spasticity, contractures, or heterotopic ossification that result in inadequate range of motion of the shoulder, elbow, wrist, fingers, hip, knee, or ankle joints in the judgment of the study physician;
- (Interventional phase only): Inability to cooperate with instructions or unwilling to commit to training sessions for 3-4 days per week over roughly 9 months;
- (Interventional phase only): A diagnosis of coronary artery disease that precludes moderate to intense exercise.

We plan to enroll 51 participants for the observational phase. 24 participants with motor incomplete SCI will continue with the interventional phase of the study. Participants will be recruited from the large population of spinal cord injury (SCI) patients followed at James J. Peters VAMC, as well as through physician referrals and IRB-approved advertising to non-veterans. The protocol will take 3 years to complete.

No conventional medication therapy or diagnostic procedures will be withheld for participation in this study. We will ask participants to refrain from undergoing additional new outpatient exercise rehabilitation programs during the duration of their involvement with the current Study.

3b. List Possible RISKS

There are several potential risks associated with placing participants in a weight-support harness for therapy involving dynamic upright sitting and standing. These include orthostatic hypotension; autonomic dysreflexia; skin breakdown; falls; and bone fractures. The potential benefits of undergoing the exercise interventions described in our protocol justify the small risk of these adverse events – similar harness-supported exercise protocols for SCI participants have been widely used and safely applied, both at the JJPVAMC (IRB-approved protocol #4843-06-090) and elsewhere (Dobkin et al. 2006; Harkema et al. 2011). We have multiple mechanisms in place to minimize these risks, as described below:

Heart rate and blood pressure will be measured at the beginning of each session and every 15 minutes thereafter. Participants with a fall in blood pressure greater than 20mm Hg systolic or 10mm Hg diastolic combined with any symptoms such as light-headedness will immediately be bought to a supine position. This is the recommendation of the Autonomic Society and the American Academy of Neurology.

Additionally, we will monitor and query participants throughout each session for symptoms of hypotension such as light-headedness or queasiness; and symptoms of autonomic dysreflexia such as severe headache, flushing, or sweating. Participants can request to end the session at any time.

Skin breakdown on harnessed areas as well as the feet will be minimized by appropriate fitting and padding, as well as close monitoring before and after each session. If a participant develops a pressure ulcer of Stage 2 or worse, training will be suspended.

To minimize the risk of fracture, participants will undergo bone density scans by dual x-ray absorptiometry (DXA) prior to participation/enrollment. Potential participants with density <3.5 SDs below the norm for lower extremity bone density will be excluded from participating in the Interventional Phase of the study. The amount of radiation exposure from the DXA scan is minimal: We estimate all of the DXA measurements combined will sum up to approximately 35 μSv of radiation exposure per scan, or 140 μSv total (**Table 4**). Four routine chest X-rays would deliver an approximate dose of 240 μSv. In addition, the average New York City resident receives approximately 300 millirem/year (which converts to 3000 μSv/year). Therefore, these participants would have to repeat all of these tests more than 20 times to receive an equivalent dose.

To minimize fall risk, all seated and standing training and assessment protocols will be conducted with participants wearing a support harness attached to an overhead unweighing system as needed. The unweighing system includes a mechanism to prevent falls of more than four inches in any direction. At least two clinicians will be at the participant's side at all times to assess comfort and provide manual assistance/stabilization as necessary.

The surface electromyography-Brain Motor Control Assessment (BMCA) protocol involves recording the endogenous electrical potentials produced by the participant's muscles using skin electrodes. No current is applied toward the participant. The procedure is analogous to an electrocardiogram. Therefore, the risks are minimal, such as transient skin irritation at the sites of surface electrode application.

In contrast to the BMCA, H-reflex testing involves the use of surface electrodes *and* exogenously applied electrical stimuli. The resulting induced signals are then recorded with surface electrodes over the back, neck, and scalp. The applied currents are small (usually less than 50 milliamperes) but may be transiently irritating or painful. Superficial muscles near the stimulating electrodes may also twitch during stimulation, but this is not physically painful or dangerous. There is no risk of seizure from H-reflex stimulation.

Evoking motor potentials using transcranial magnetic stimulation (TMS) carries several potential risks. Most of these risks are much greater during application of *repetitive* TMS, which will *not* be conducted in this study. We will be using a MagPro R30 device (MagVenture) for diagnostic (not repetitive) TMS. This device has FDA 510K clearance (approval #K091940). The most serious risk of TMS is induction of seizures. TMS-induced seizures are usually focal, but in some cases can become generalized. To minimize this risk, participants with underlying brain injury that increases the risk of TMS-induced seizures will be excluded from participation. Additionally, participants taking medications that significantly lower seizure thresholds, such as anti-psychotics, will be excluded. Furthermore, the applied stimulus intensity will be kept below 200% of the motor threshold for each muscle. This intensity and single-pulse frequency fall far below the recommended safe guidelines delineated by an international workshop on TMS safety (Rossi et al. 2009). Furthermore, participants with implanted devices with electromagnetic properties, such as spine stimulators, deep brain stimulators, vagal nerve stimulators, cardiac pacemakers, cochlear implants, or aneurysm clips, will be excluded. The co-investigator who will be performing the TMS protocol (Noam Harel) is a neurologist experienced in treating seizures. Note that this use of TMS does *not* involve *repetitive* TMS. Repetitive TMS is a separate entity utilized to achieve modulation of brain activity. Even though it is considered to be higher-risk than diagnostic/single pulse TMS, repetitive TMS has been FDA-approved for treatment of refractory major depression. This repetitive TMS device (NeuroStar) is in clinical use at the JJPVAMC Department of Psychiatry (FDA 510K approval #K083538).

TMS pulses generate loud auditory clicks. Hence, all participants will wear earplugs during the procedure. TMS may also cause scalp tingling sensations or pain that is almost always mild and transient. This occurs approximately half as frequently in participants exposed to sham-stimulation. This

is much less common using single-pulse TMS than repetitive TMS. Our protocol will use only single-pulse TMS.

We also point out that a repetitive TMS device has been FDA-approved for treating patients with major depression. One of these devices (Neurostar by Neuronetics) is already in use within the psychiatry department at JJPVAMC. Our proposed protocol will use a research TMS device (MagPro R30) that will deliver single but not repetitive pulses. To repeat, the use of only single pulse TMS, as well as all the other precautions and exclusion criteria we will follow in our TMS protocol, far exceed the recommended guidelines established by Rossi et al.

There is a small chance that subjects may feel uncomfortable or distressed by some survey questions about pain, spasticity, or quality of life. We will emphasize the voluntary nature of answering any questions. An MD will be on the premises if distressed subjects need further evaluation or endorse suicidal thoughts, and immediate referral to a mental health provider will be arranged if necessary On the SCI-QoL questionnaire, endorsement of suicidal thoughts would qualify at a level of 3 (3=sometimes) or above (4=often, 5=always).

<u>Protection against Risk</u>

Most of the information that participants provide will not be identifiable. Participant data results will be stored on the VA network in a password-protected file. No identifiable information will be linked to this file. The study team members will have a separate file of participant contact information, also stored on the VA network. Participants will be assured that any “hard copies” of their contact information or data will be kept in a securely locked cabinet in a locked private office. This data will not be destroyed. There is minimal risk of a breach of confidentiality or data security.

Study coordinators and research assistants will be trained to conduct all procedures in a safe and effective manner that produces reproducible results. Study personnel with experience working with SCI participants will supervise all sessions. All staff will have undergone the appropriate training to use the equipment properly and safely. The study personnel will refer participants to the proper medical or psychological resources for any identified conditions or problems as a consequence of the research.

In the event of a serious adverse event, it will be reported to the IRB within 24 hours and study interventions will be discontinued until the study physician states that it is safe to resume the study. Adverse events will be recorded in a data sheet and reported annually to the IRB. When appropriate, necessary medical or professional intervention will be provided for any serious or regular adverse event warranting treatment.

A formal Data Safety Monitoring Board will not be needed for this study.

Provisions for keeping data confidential are established. All electronic data will be kept on the secure VA network. All intake forms are de-identified according to HIPAA regulations. Consent forms, along with any other forms containing identifiable information, are kept in a locked filing cabinet. Only members of the investigative team will be able to use the information.

Any unexpected complications that may occur will be discussed with the study physician and/or the participant’s SCI physician. Dr. Noam Y. Harel (PI), a neurologist, will serve as the study physician. He will be present and directly involved in most sessions. Dr. Ann M. Spungen (co-I), an applied physiologist and exercise physiologist, will review each participant’s exercise training progress. In the event of a serious adverse event, it will be reported to the IRB within 24 hours, and study interventions will be discontinued until the study physician states that it is safe to resume the study. Adverse events will be recorded in a data sheet and reported annually to the IRB.

3c. Possible Benefits

If eligible for the Interventional Phase of the study, participants in this study could potentially achieve neurological and general health benefits. Neurologically, they may show better balance, better leg strength, improved spasticity, and better mobility. As for general health, participants may show increased cardiovascular endurance, decreased fatigue, improved orthostatic tolerance, or any of the numerous

other physical and mental benefits of exercise training that could lead to improved quality of life. Participants will be reminded that none of these benefits are guaranteed. In addition to the possibility of direct benefits obtained by the study participants, the entire population of persons with paraplegia may benefit from the knowledge gained regarding the effects of combined hand and balance rehabilitation therapy compared to conventional locomotor therapy for paraplegia.

The potential benefits should outweigh the risks of performing our study, especially with the extensive risk reduction strategies described above.

4. PREVIOUS WORK DONE BY YOU OR YOUR COLLABORATORS ON THIS OR RELATED PROJECTS, LISTING PUBLICATIONS.

Related projects at the SCI Center of Excellence

Two ongoing IRB protocols within the COESCI encompass many similar training and assessment measures, as well as safety techniques, as those described in this protocol. One of these is the ReWalk Study (protocol number SPU-09-011), and the other is the Lokomat Study (protocol number 4843-06-090). Each of these protocols are done in collaboration with both the SCI Service and Rehabilitation Service investigating the benefits of using the ReWalk or the Lokomat as a rehabilitation tool for persons with chronic SCI. In fact, Lokomat training will be used in the current protocol as the ‘conventional’ training technique. Body weight-supported treadmill training has shown numerous benefits in patients with various pathologies such as SCI, Stroke and Multiple Sclerosis (MS) for gait training.

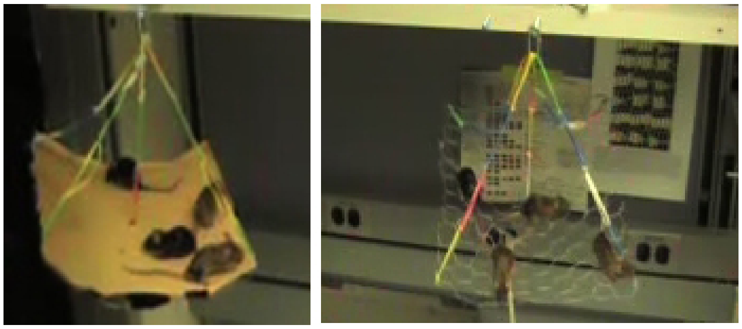

Fig 5 – BRAINSTEM-ONLY apparatus (left); MULTIMODAL (Brainstem+CST) apparatus (right).

Mouse multimodal training regimen stimulating cortical and brainstem circuits

Initial work toward the goals of this proposal was performed in mouse models of SCI. We designed a training regimen to simultaneously invoke skilled forelimb use (CST) and postural reflexes (vestibulospinal, reticulospinal, and other brainstem tracts) by requiring mice to grip and maintain balance on a mobile wire mesh. A typical setup is shown in **Figure 5**. The platforms are suspended by an array of elastic bands that results in a continually shifting plane of motion as mice ambulate. This stimulates vestibular and other brainstem tracts involved in postural control. The BRAINSTEM-ONLY platform has a smooth surface, whereas the MULTIMODAL platform consists of a fine wire grating. The wire grating stimulates CST circuits as mice assimilate tactile and visual information to plan and execute forelimb movements to grasp the wires. The elastic suspensions were adjusted to result in the same amount of angular displacement on either the BRAINSTEM-ONLY or MULTIMODAL platforms. By fixing the grating to a stationary surface, the platform becomes a CST-ONLY training apparatus.

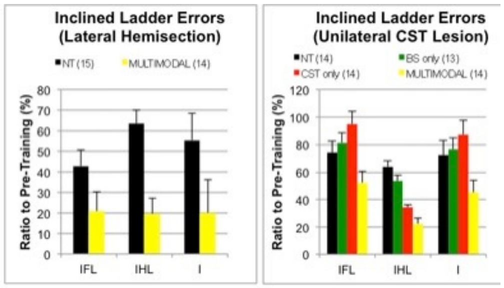

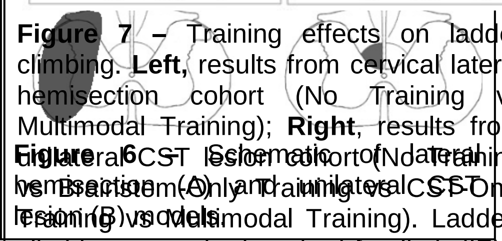

Figure 7 – Training effects on ladder climbing. **Left,** results from cervical lateral hemisection cohort (No Training vs Multimodal Training); **Right**, results from unilateral CST lesion cohort (No Training vs Brainstem-Only Training vs CST-Only Training vs Multimodal Training). Ladder-climbing errors by impaired forelimb (IFL), impaired hindlimb (IHL), or both impaired limbs (I), relative to pre-training. MULTIMODAL training (BS+CST) reduces errors made by impaired limbs.

Figure 6 – Schematic of lateral hemisection (A) and unilateral CST lesion (B) models

Multimodal training improves behavioral outcomes in two different models of mouse central nervous system injury

The training approach described above was applied in cohorts of mice that had undergone two distinct forms of incomplete central nervous system injury: one cohort underwent lateral cervical hemisection; the other underwent unilateral CST lesion (**Fig 6**). Whereas the cervical hemisection model spared most CST fibers, the unilateral

CST lesion focused exclusively on CST fibers. Both of these injury models allowed us to study both forelimb and hindlimb recovery, as well as to compare recovery of affected to unaffected sides (Harel et al. 2010; Harel et al. 2013).

In the cervical hemisection experiment, training with the MULTIMODAL apparatus was compared to no training. In the unilateral CST lesion mice, all four training groups were compared. In both lesion models, mice in the MULTIMODAL training group demonstrated improved performance in inclined grid walking. Interestingly, the magnitude of the training effect was higher for the impaired hindlimb than the impaired forelimb **(Figure 7)**. However, performance did not improve in other behavioral tests that were not specific to the training task, such as Rotarod endurance, cylinder exploration, and open-field ambulation (not shown). This lack of broader overall benefit is consistent with task-specific benefits conferred by training in a variety of animal models and human patients with stroke and SCI (Garcia-Alias et al. 2009; Grasso et al. 2004; Magnuson et al. 2009; Smith et al. 2006).

Anatomically, mice that underwent cervical lateral hemisection demonstrated increased growth of serotonergic fibers on the lesioned side, but there was no difference among training groups. Histological analysis in the unilateral CST lesion cohort is ongoing. This model allows testing of two distinct mechanisms of recovery – the ability of the *injured* CST to form detour connections with brainstem circuits *above* the lesion; and the ability of the *uninjured* CST to form detour connections with segmental spinal neurons *below* the lesion.

5. SIGNIFICANCE OF THIS RESEARCH.

The information obtained in this study may be useful scientifically to others. The battery of functional and electrophysiological tests could provide a model for defining partially spared neural circuitry in SCI patients. This information would allow rehabilitation interventions to more directly target these spared circuits. If our novel exercise intervention shows improved benefits over standard training, then SCI patients and their clinicians elsewhere should be able to relatively easily adopt these techniques. If the intervention does not show improved benefits, then there are many parameters that could be systematically tested, by us and/or others, to attempt to improve outcomes.

LIST OF KEY BIBLIOGRAPHIC REFERENCES.

Adams, M.M., Ginis, K.A.M. & Hicks, A.L., 2007. The spinal cord injury spasticity evaluation tool: development and evaluation. *Archives of physical medicine and rehabilitation*, 88(9), pp.1185–92.

Bareyre, F.M. et al., 2004. The injured spinal cord spontaneously forms a new intraspinal circuit in adult rats. *Nat Neurosci*, 7(3), pp.269–277.

Benz, E.N. et al., 2005. A physiologically based clinical measure for spastic reflexes in spinal cord injury. *Archives of physical medicine and rehabilitation*, 86(1), pp.52–9.

Brogardh, C. et al., 2010. Mode of hand training determines cortical reorganisation: a randomized controlled study in healthy adults. *J Rehabil Med*, 42(8), pp.789–794.

Brus-Ramer, M. et al., 2007. Electrical stimulation of spared corticospinal axons augments connections with ipsilateral spinal motor circuits after injury. *J Neurosci*, 27(50), pp.13793–13801.

Carmel, J.B. et al., 2010. Chronic electrical stimulation of the intact corticospinal system after unilateral injury restores skilled locomotor control and promotes spinal axon outgrowth. *J Neurosci*, 30(32), pp.10918–10926.

Courtine, G. et al., 2008. Recovery of supraspinal control of stepping via indirect propriospinal relay connections after spinal cord injury. *Nat Med*, 14(1), pp.69–74.

Courtine, G. et al., 2009. Transformation of nonfunctional spinal circuits into functional states after the loss of brain input. *Nat Neurosci*, 12(10), pp.1333–1342.

Ditunno Jr., J.F. et al., 2007. Validity of the walking scale for spinal cord injury and other domains of function in a multicenter clinical trial. *Neurorehabil Neural Repair*, 21(6), pp.539–550.

Dobkin, B. et al., 2006. Weight-supported treadmill vs over-ground training for walking after acute incomplete SCI. *Neurology*, 66(4), pp.484–493.

Garcia-Alias, G. et al., 2009. Chondroitinase ABC treatment opens a window of opportunity for task-specific rehabilitation. *Nat Neurosci*, 12(9), pp.1145–1151.

Grasso, R. et al., 2004. Recovery of forward stepping in spinal cord injured patients does not transfer to untrained backward stepping. *Exp Brain Res*, 157(3), pp.377–382.

Harel, N.Y. et al., 2013. Multimodal exercises simultaneously stimulating cortical and brainstem pathways after unilateral corticospinal lesion. *Brain Research*.

Harel, N.Y. et al., 2010. Nogo Receptor Deletion and Multimodal Exercise Improve Distinct Aspects of Recovery in Cervical Spinal Cord Injury. *J Neurotrauma*, 27(11), pp.2055–2066.

Harkema, S. et al., 2011. Effect of epidural stimulation of the lumbosacral spinal cord on voluntary movement, standing, and assisted stepping after motor complete paraplegia: a case study. *Lancet*, Early onli.

Hayes, K.C. & Kakulas, B.A., 1997. Neuropathology of human spinal cord injury sustained in sports-related activities. *J Neurotrauma*, 14(4), pp.235–248.

Hebb, D.O., 1949. *The organization of behavior; a neuropsychological theory*, New York,: Wiley.

Iles, J.F., 1996. Evidence for cutaneous and corticospinal modulation of presynaptic inhibition of Ia afferents from the human lower limb. *The Journal of physiology*, 491 (Pt 1, pp.197–207.

Jankowska, E. & Edgley, S.A., 2006. How can corticospinal tract neurons contribute to ipsilateral movements? A question with implications for recovery of motor functions. *Neuroscientist*, 12(1), pp.67–79.

Jensen, J.L., Marstrand, P.C. & Nielsen, J.B., 2005. Motor skill training and strength training are associated with different plastic changes in the central nervous system. *J Appl Physiol*, 99(4), pp.1558–1568.

Kakulas, B.A., 1987. The clinical neuropathology of spinal cord injury. A guide to the future. *Paraplegia*, 25(3), pp.212–216.

Kim, J.H., Chung, Y.J. & Shin, H.K., 2010. Effects of Balance Training on Patients with Spinal Cord Injury. *J Phys Ther Sci*, 22, pp.311–316.

Klimstra, M. & Zehr, E.P., 2008. A sigmoid function is the best fit for the ascending limb of the Hoffmann reflex recruitment curve. *Exp Brain Res*, 186(1), pp.93–105.

Knikou, M. & Mummidisetty, C.K., 2011. Reduced reciprocal inhibition during assisted stepping in human spinal cord injury. *Experimental neurology*, 231(1), pp.104–12.

Lee, D.C. et al., 2004. Toward an objective interpretation of surface EMG patterns: a voluntary response index (VRI). *J Electromyogr Kinesiol*, 14(3), pp.379–388.

Lemon, R.N. & Griffiths, J., 2005. Comparing the function of the corticospinal system in different species: organizational differences for motor specialization? *Muscle Nerve*, 32(3), pp.261–279.

Magnuson, D.S. et al., 2009. Swimming as a model of task-specific locomotor retraining after spinal cord injury in the rat. *Neurorehabil Neural Repair*, 23(6), pp.535–545.

Matsuyama, K. et al., 2004. Locomotor role of the corticoreticular-reticulospinal-spinal interneuronal system. *Progress in brain research*, 143(03), pp.239–49.

McKay, W.B. et al., 2004. Clinical neurophysiological assessment of residual motor control in post-spinal cord injury paralysis. *Neurorehabilitation and neural repair*, 18(3), pp.144–53.

McKay, W.B. et al., 2010. Neurophysiological characterization of motor recovery in acute spinal cord injury. *Spinal Cord*, 49(3), pp.421–429.

Minassian, K. et al., 2007. Human lumbar cord circuitries can be activated by extrinsic tonic input to generate locomotor-like activity. *Hum Mov Sci*, 26(2), pp.275–295.

Morita, H. et al., 2001. Modulation of presynaptic inhibition and disynaptic reciprocal Ia inhibition during voluntary movement in spasticity. *Brain*, 124(Pt 4), pp.826–837.

Nathan, P.W., Smith, M. & Deacon, P., 1996. Vestibulospinal, reticulospinal and descending propriospinal nerve fibres in man. *Brain*, 119 (Pt 6, pp.1809–1833.

NSCISC, N.S.C.I.S.C.-, 2013. Spinal cord injury facts and figures at a glance. *The journal of spinal cord medicine*, 36(5), pp.568–9.

Organization, W.H., 1998. Development of the World Health Organization WHOQOL-BREF quality of life assessment. The WHOQOL Group. *Psychological medicine*, 28(3), pp.551–8.

Perez, M.A. et al., 2004. Motor skill training induces changes in the excitability of the leg cortical area in healthy humans. *Exp Brain Res*, 159(2), pp.197–205.

Pettersson, L.G. et al., 2007. Skilled digit movements in feline and primate--recovery after selective spinal cord lesions. *Acta Physiol (Oxf)*, 189(2), pp.141–154.

Roche, N. et al., 2011. Effects of anodal transcranial direct current stimulation over the leg motor area on lumbar spinal network excitability in healthy subjects. *J Physiol*, 589(Pt 11), pp.2813–2826.

Rossi, S. et al., 2009. Safety, ethical considerations, and application guidelines for the use of transcranial magnetic stimulation in clinical practice and research. *Clinical neurophysiology : official journal of the International Federation of Clinical Neurophysiology*, 120(12), pp.2008–39.

Sadowsky, C.L. & McDonald, J.W., 2009. Activity-based restorative therapies: concepts and applications in spinal cord injury-related neurorehabilitation. *Dev Disabil Res Rev*, 15(2), pp.112–116.

Serranova, T. et al., 2008. Abnormal corticospinal tract modulation of the soleus H reflex in patients with pure spastic paraparesis. *Neurosci Lett*, 437(1), pp.15–19.

Sherwood, A.M., Dimitrijevic, M.R. & McKay, W.B., 1992. Evidence of subclinical brain influence in clinically complete spinal cord injury: discomplete SCI. *J Neurol Sci*, 110(1-2), pp.90–98.

Smith, R.R. et al., 2006. Effects of swimming on functional recovery after incomplete spinal cord injury in rats. *Journal of neurotrauma*, 23(6), pp.908–19.

Thomas, S.L. & Gorassini, M.A., 2005. Increases in corticospinal tract function by treadmill training after incomplete spinal cord injury. *J Neurophysiol*, 94(4), pp.2844–2855.

Trimble, M.H. et al., 2001. Acute effects of locomotor training on overground walking speed and H-reflex modulation in individuals with incomplete spinal cord injury. *J Spinal Cord Med*, 24(2), pp.74–80.

Tulsky, D.S. et al., 2011. Developing a contemporary patient-reported outcomes measure for spinal cord injury. *Archives of physical medicine and rehabilitation*, 92(10 Suppl), pp.S44–51.

Wellek, S. & Blettner, M., 2012. On the proper use of the crossover design in clinical trials: part 18 of a series on evaluation of scientific publications. *Deutsches Ärzteblatt international*, 109(15), pp.276–81.
